# Supplementary material for: Age-related modulations of alpha and gamma brain activities underlying anticipation and distraction
Source: PLoS One. 2020 Mar 12;15(3):e0229334. doi: 10.1371/journal.pone.0229334 (PMC7067396; doi:10.1371/journal.pone.0229334)
Supplement: S2 Fig — (DOCX) [file pone.0229334.s002.docx]

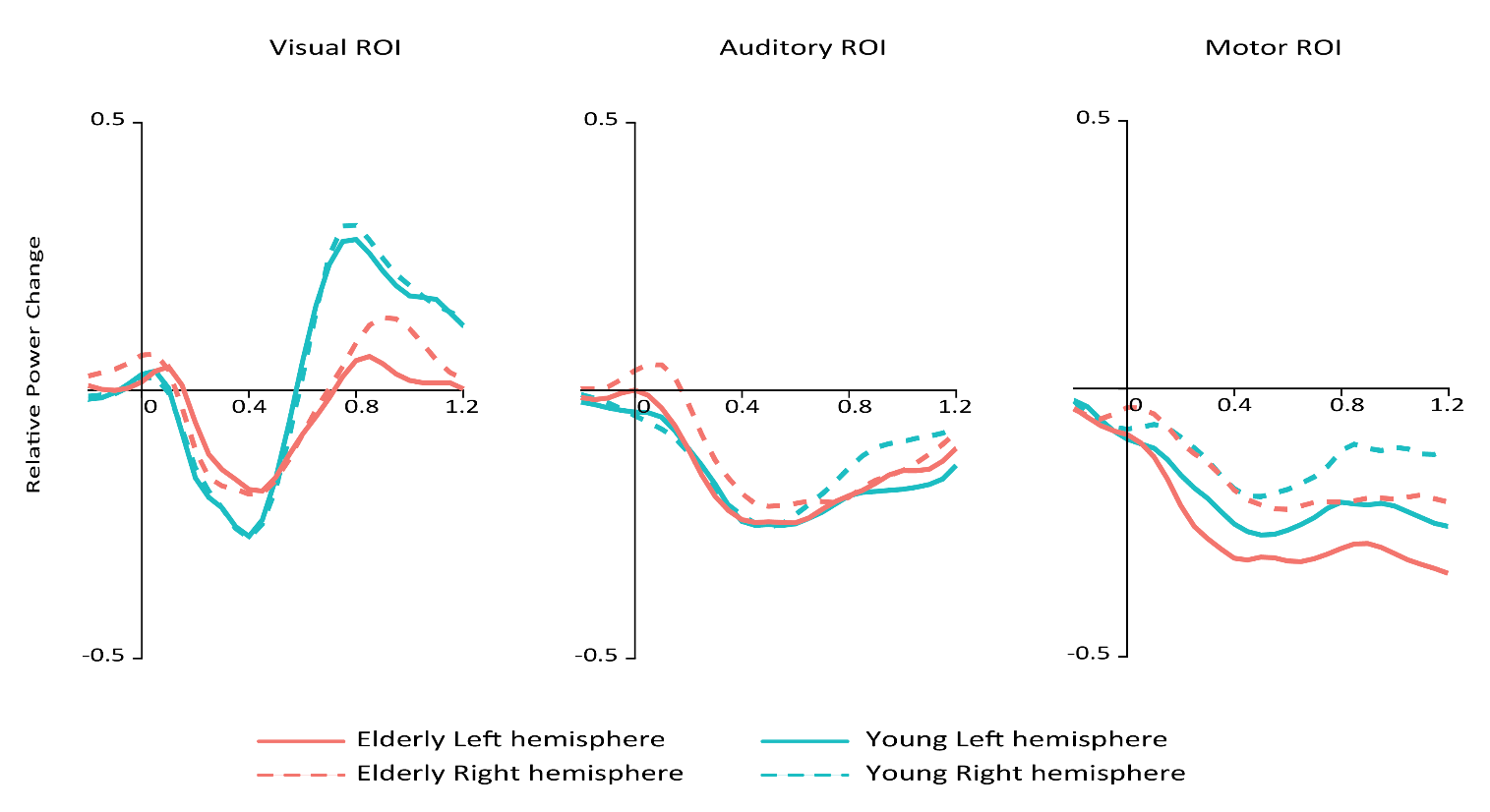


**Supplementary Figure 2**: Time-course of mean alpha power centered on alpha-peak frequency for each region and each participant.
