# Supplementary material for: Age-related modulations of alpha and gamma brain activities underlying anticipation and distraction
Source: PLoS One. 2020 Mar 12;15(3):e0229334. doi: 10.1371/journal.pone.0229334 (PMC7067396; doi:10.1371/journal.pone.0229334)
Supplement: S3 Fig — (DOCX) [file pone.0229334.s003.docx]

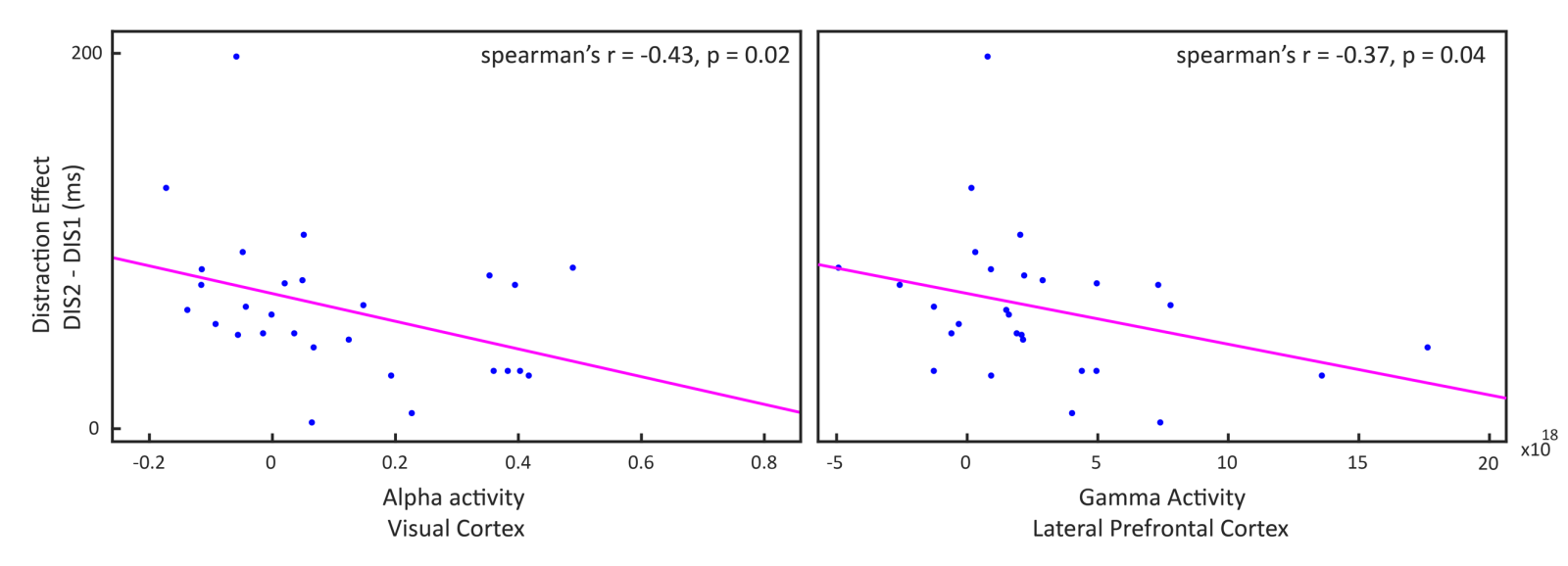


**Supplementary Figure 3**: Scatter plots of behavioral distraction effect against alpha activity in the visual cortex and gamma activity in the lateral prefrontal cortex.
