# Supplementary material for: Age-related modulations of alpha and gamma brain activities underlying anticipation and distraction
Source: PLoS One. 2020 Mar 12;15(3):e0229334. doi: 10.1371/journal.pone.0229334 (PMC7067396; doi:10.1371/journal.pone.0229334)
Supplement: S4 Fig — (DOCX) [file pone.0229334.s004.docx]

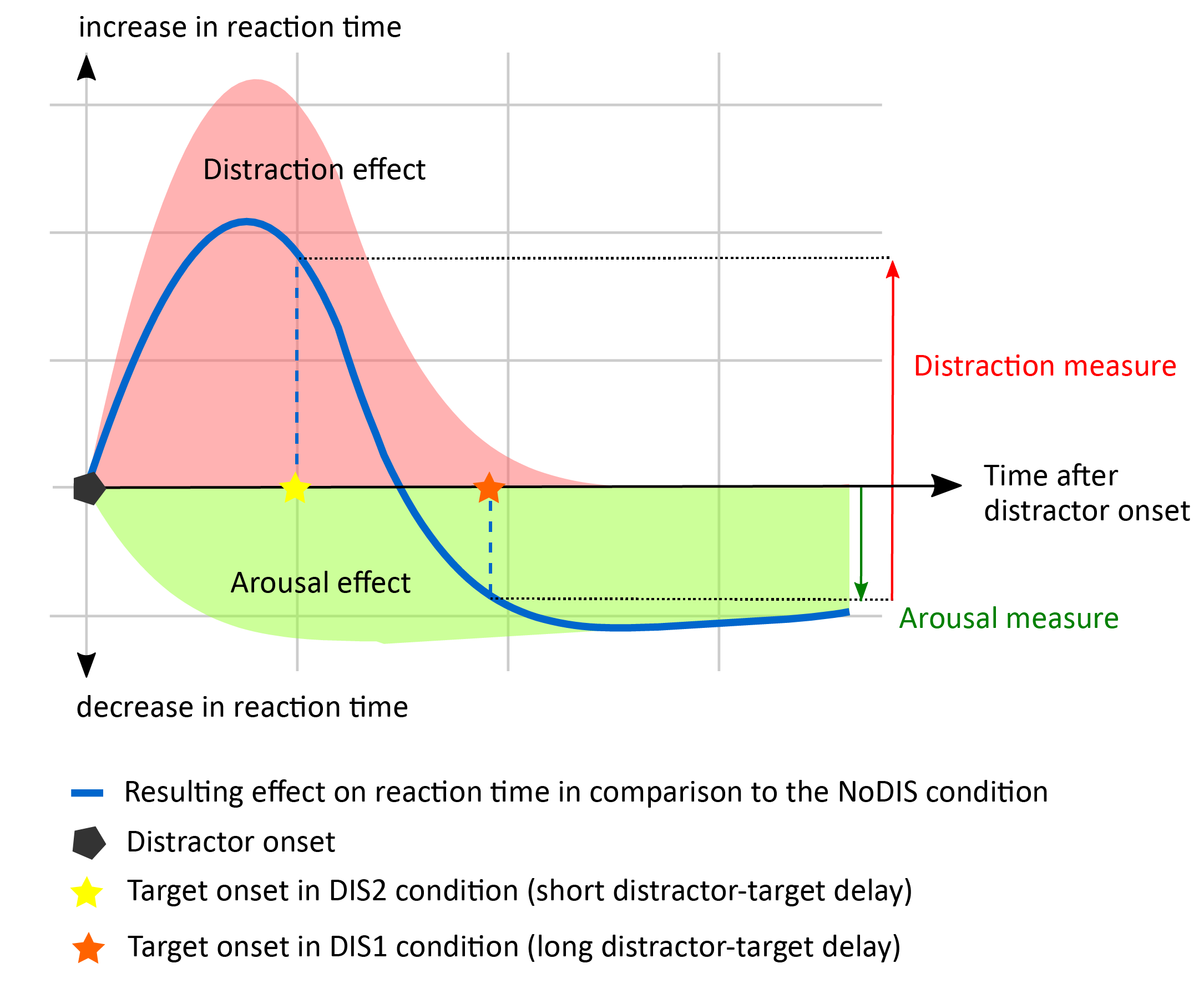


**Supplementary Figure 4**: Schematic representation of the behavioral effects of distracting sounds in the Competitive Attention Test.
