# Supplementary material for: Age-related modulations of alpha and gamma brain activities underlying anticipation and distraction
Source: PLoS One. 2020 Mar 12;15(3):e0229334. doi: 10.1371/journal.pone.0229334 (PMC7067396; doi:10.1371/journal.pone.0229334)
Supplement: Attachment — Submitted filename: elshafei et al Response to Reviewers.docx [file pone.0229334.s006.docx]

We thank the editor and reviewers for their constructive comments on our manuscript now entitled "Age-Related Modulations of Alpha and Gamma Brain Activities Underlying Anticipation and Distraction" and for the opportunity to respond. In section ONE, we address a main concern shared by both reviewers: the analysis of the alpha band (R1.1.a, R2.1, R2.2). Both reviewers recommended the adoption of a data-driven approach to our alpha-band analysis using DICS beamformer, similar to the gamma band analysis. This implied major reorganization of the analysis pipeline for alpha and gamma band activities to make them as similar as possible. It’s important to note that these changes in the methods did not alter the main findings of the study. In section TWO, we have laid out the reviewers’ comments (in green font) along with our replies in black. We have taken the liberty to number the reviewer’s comments so that they are easier to refer to. In the revised manuscript, modifications are highlighted in yellow.

**SECTION ONE: COMMON CONCERNS**

**Figure 1.** Summary of the updated analysis pipeline.

In the following is a summary of the new analyses we have run to answer reviewers’ comments (see Figure1). Detailed analysis and results have been included in the revised version of the manuscript (all points except 3a) and in supplementary data (point 3a).

**I. Alpha band analysis:**

**[1] On the sensor level:**

For each group, activity of interest (between 0 and 1.2s post-cue and 5-45Hz: Figure 2) was contrasted to baseline (-0.6 to -0.2s pre-cue) using Cluster-Based Permutation tests. One can see that, in comparison to the baseline, both young and elderly participants present a decrease in power in the alpha band from 0.2 to 1.2 s after cue onset. However, only the young participants display an increase in alpha power occurring from 0.6 to 1.2 s after cue onset within the high alpha band (11-15Hz). These findings in the young participants replicate our previous findings in another group of young adults (ElShafei et al., 2018, eNeuro).

[**2a] On the source level**:

For each group, post-cue (0.6-1s) low (7-11Hz) and high (11-15Hz) alpha source activity were contrasted against baseline (-0.6 to -0.2s) using DICS beamformer and Cluster-Based Permutation tests (see Figure 3). In young controls, we found a decrease (compared to baseline) in low alpha power in auditory cortices and in fronto-parietal regions with a maximum around the left motor cortices; and an increase in high alpha power in occipital areas. Once again, these findings in the young participants are in agreement with our previous findings in another group of young adults (ElShafei et al., 2018, eNeuro). In elderly participants, we found similar results in the low alpha band. However, in the high alpha band, we found no power increase, but a power decrease in frontal and motor areas.

[**2b**] **On the source level**:

We have contrasted the aforementioned post-cue activity in the low and high alpha bands between groups, using DICS beamformer and Cluster-Based Permutation Tests. No significant difference was found in the low alpha band. In the high alpha band, we found a significant higher power in young participants, predominantly in occipital and left motor areas (Figure 3).

**[3a] On the virtual electrode level**:

In order to further appreciate the specificity of our results to the low and high alpha bands, we have plotted the time-frequency distributions of power in our ROIs. The activity distribution is quite different across ROIs and only the younger participants display an increase in the high alpha band in the occipital regions (Figure 4).

**[3b]** **On the virtual electrode level**:

We have added the factor hemisphere to the analysis of the alpha peak frequency. We also have run an additional analysis to investigate the impact of the cue information on alpha power based on the individual alpha peak frequency in each ROI.

**II. Gamma band analysis:**

[**4] On the sensor level:**

For each group, activity of interest (between 0 and 350ms post-dis and 50-110 Hz: Figure 5) was contrasted to the activity to fake distractor in each group using Cluster-Based Permutation tests. One can see that both young and elderly participants present an increase in power between 100 and 300 ms in the 60-100 Hz frequency band.

**[5] On the source level**:

For each group, post-dis (100-300 ms) gamma (60-100Hz) source activity was contrasted against fake distractor activity, using DICS beamformer and Cluster-Based Permutation tests. In both groups, we found an increase in gamma power in the auditory cortices, the temporo-parietal junctions and the ventro-lateral PFC (Figure 6A&C)

**[6]** **On the source level:**

We have run an additional analysis to investigate the impact of the cue information on gamma power in each group. Young participants presented an increase in gamma power in median frontal regions for informative cue compared to uninformative cue, while no significant difference was found in elderly (Figure 6D).

**Figure 2**. **Sensor level alpha activity.** Comparison between post-cue activity of interest (between 0 and 1.2s post-cue and 5-45Hz) to baseline (-0.6 to -0.2s pre-cue) for young and elderly participants. **A-B.** Topographical maps represent T-values, masked at p < 0.1, for the comparison in the alpha band (7-15 Hz and 0.6-1s post-cue). **C-D.** Time-frequency representations depict T-values, of the same tests, masked at p<0.1 and averaged across sensors highlighted by black boxes on the corresponding topographical maps.

**Figure 3**. **Source level alpha activity.** **A-B.** Distributions of T-values, masked at p < 0.05, from cluster-based permutation tests contrasting time-frequency windows of interest (low and high alpha) against baseline activity at the source level for each group. **C**. Distributions of T-values, masked at p < 0.05, from cluster-based permutation tests contrasting baseline-corrected time-frequency window of interest between groups.

**Figure 4**. Time-frequency representations of power baseline corrected (-600 to -200ms pre-cue onset) for each group and ROI.

**Figure 5**. **Sensor level gamma activity.** **Upper row**: Topographical maps averaged between 50-110 Hz and 0-0.35 seconds post-distractor onset, of the T-values, masked at p < 0.05 of the contrast between distractor and surrogate distractor gamma activity for each group. **Lower row**: Time-frequency representations of T-values (of the aforementioned test) of the sensors highlighted by red boxes. Note that each TF plot is surrounded by the frequency distribution of T-values of the aforementioned sensors averaged across the time dimension and by the time distribution of t-values of the aforementioned sensors averaged across the frequency dimension.

**Figure 6.** **Source level gamma activity.** **A&C**. Distributions of T-values, masked at p<0.05, from Cluster Based Permutation tests contrasting real and surrogate distractor gamma activity (60-100Hz and 0.1-0.3 post-distractor) for elderly and young groups, respectively, at the source level. **B**. Between group comparison of surrogate-corrected distractor gamma activity (60-100Hz and 0.1-0.3 post-distractor) at the source level. Positive T-values indicate stronger gamma activity in the young group. **D.** Between cue condition comparison of surrogate-corrected gamma activity to distractor in the young group. Positive T-values indicate stronger gamma activity in the informative cue condition. Note that no significant effect was found in the elderly group.

**SECTION TWO: SPECIFIC CONCERNS**

**Reviewer #1:**

**The authors of this manuscript describe an MEG study that aims to investigate age-related differences in auditory top-down and bottom-up processes. The authors show differences in alpha power between younger and older adults that are interpreted to reflect top-down processes. They also find differences in gamma-band activity between age groups that are interpreted to reflect bottom-up processes. The topic is interesting, and the study could be received well by the community. However, I have some concerns regarding the data analysis and interpretation of the data, described in detail below:**

**Alpha power analysis:**

**R1.1a. The alpha-power analysis and data presentation appear selective and not fully straight forward. The authors use a time-domain spatial filter (LCMV) to project data into source/virtual space and then analyze data in the frequency domain. That is, the filter may be based on very different, non-alpha activity, aspects of the data. It is not clear how this affects the results. For the gamma band they use, more appropriately, a spatial filter for frequency data (DICS). It is not clear why two different approaches were chosen, because the DICS beamformer would have been the appropriate choice also for the alpha data. I recommend making the data analysis consistent and using a DICS beamformer throughout. Moreover, despite focusing on a specific frequency band (alpha), the authors still want to show full time frequency plots from the wavelet analysis, similar to what they have done for the gamma band. The reader wants to see how power spreads across frequencies, which also helps to get an idea about differences in alpha frequency. Moreover, full source localizations for the alpha activity should be provided. From the plots in Figure 5, it is not sufficiently clear that there are actually multiple different sources. It looks like there may also be effects of volume conduction present. Furthermore, it was not clear why the alpha band was divided into fairly wide sub-bands. It is very uncommon to go up to 15 Hz, and this would need to be justified. The differences in alpha frequency may further impact effects of alpha power, using a broad frequency window, because the frequency window chosen may not appropriately reflect power, if there indeed different sources with different best frequencies. In short, the authors want to show time-frequency plots for alpha data, use the DICS beamformer for alpha activity, and show full source localizations for alpha power. This should be no problem, given that the authors used this approach for the gamma band.**

We thank the Reviewer for bringing up this concern, it seems that our methods have appeared selective or not straight forward. The alpha-power analyses were based upon strong hypotheses regarding regions of interest (ROIs: visual and auditory) and frequency bands (low and high) where alpha activity would be modulated during our task. These hypotheses were based upon a previous study using a similar paradigm in a different group of young participants (ElShafei et al., 2018 eNeuro). One aim of the current study was to highlight how alpha power is modulated by the age in these ROIs. For this purpose, we chose the LCMV approach which allows for the reconstruction of time courses of activity at each voxel. We are not sure to understand the point raised by the reviewer concerning the use of the LCMV approach. The LCMV approach does not test the whole brain emergence of alpha activity. Nevertheless, the spectral content of the data in a certain frequency band would not change as long as that band has been included for the calculation of the covariance matrix. In other words, the LCMV filters are calculated using the leadfield matrices and the data covariance matrix (computed for all frequencies).

However, as detailed at the beginning of this document, we have followed the reviewer comment and reanalyzed the alpha activity using a DICS beamformer approach. These additional analysis show that low and high alpha power are source localized in different brain regions confirming both differences in alpha frequencies and multiple distinct sources. In young participants, it seems difficult to explain the increase in high alpha power in occipital regions and the decrease in low alpha power over motor and auditory areas by volume conduction. The distinct time-frequency distributions (Figure 2) and time-profiles (Figure 4) also argue for dissociated sources and alpha bands.

**R1.1b. Finally, the authors, in several places of the manuscript, mention that for auditory cortex there is an alpha desynchronization whereas for visual cortex there is alpha synchronization. There is no reason, based on what the time courses look like, to assume that there are functional differences. A reduction from baseline present is present in all regions, just that the increase in power happens to become positive in a later time window for the visual region. Some of the differences could come from volume conduction. The authors want to tune down interpretations and wording in this regard (e.g., it reads oddly in line 331 that power was lower for older compared to younger adults for two contrasts, but that one was referred to as synchronization and one as desynchronization; this has also implications do the interpretation of facilitation and suppression).**

In our previous study (ElShafei et al., 2018, eNeuro), we interpreted our findings in light of the rather well-established inhibition-timing and gating-by-inhibition hypotheses, i.e. we regard a decrease in alpha power relative to a baseline as a marker of an increases excitability in underlying cortical tissue; while an alpha power increase relative to a baseline would index a decrease in cortical excitability. We agree that, in occipital regions, we first observe a decrease in alpha power, most likely reflecting the processing of the visual cue. However, in occipital regions, this decrease is followed by an increase in alpha power significantly different from the baseline level. As mentioned above, time-frequency distributions (Figure 2) and time-profiles (Figure 4) of alpha power in auditory, motor and visual areas are quite different, arguing against volume conduction effects.

Therefore, we interpret an increase in alpha power as a decrease in cortical excitability resulting from a top-down suppressive mechanisms. We have better specify this point in the Discussion.

**R1.2a. The separation into an early and late time window during which a distractor may occur could be motivated more strongly. In fact, the authors make a clear distinction about the processes that happen for early vs. late distractors. I am not convinced that this is necessarily the case and I also do not understand how they came up with the processes they attribute to the early and late time window. Assuming the distractors were uniformly spaced in time (the authors want to describe more explicitly how distractors were spaced), the data could also be analyzed more continuously, e.g., fitting a linear function to the RT data as a function of distractor time. The choice of separating an early and late time window is a methodological choice that may impact interpretation here. It may actually be that RTs “just” increase with increasing time between cue and distractor. This would suggest one process, not two.**

We apologize for the lack of clarity regarding the uniformity of the distractors in time. Distractors were indeed uniformly paced in time and this is now explicitly mentioned in the manuscript. We would like to clarify our view on the impact of distracting sounds on reaction times. When claiming that distracting sounds trigger two phenomena, we do not only compare early and late distractor conditions, we also take into account the condition with no distracting sound. If distracting sounds only trigger attentional capture leading to a cost in reaction times, participants should be slower in presence of a distracting sound, irrespective of the cue distractor delay. However, participants are faster after early distracting sounds than in the absence of distracting sounds. This behavioral pattern has been observed in several independent studies using a similar paradigm (Bidet-Caulet et al., 2015, Masson and Bidet-Caulet 2018, ElShafei et al., 2019, Hoyer et al., bioRxiv).

In their 2018 study, Masson and Bidet-Caulet demonstrated that, regardless of the sensory modality, an unexpected distracting sound would induce a long-lasting (1500ms) facilitation effect (i.e. decrease in reaction times) whose neural markers (P3a) correlated with subjective arousal ratings of the distracting sounds. On top of this arousal effect, there is a stronger, yet more short-lived detrimental attentional capture effect. This double interpretation of the impact of distracting sounds on reaction times has also been proposed by other research groups (SanMiguel et al., 2010b, 2010a; Wetzel et al., 2013, 2012; Parmentier et al., 2010; Näätänen, 1992). Based upon these studies, the net effect of an irrelevant unexpected stimulus depends on whether this distractor would occur early enough that the detrimental attentional capture effect would wear off when the target appears, and the beneficial effect of arousal could manifest as faster reaction times.

As suggested by the reviewer, we plotted RT as a function of the cue-distractor delay (see figure 6 below). We performed Bayesian Kendall’s Tau correlations on the first 5 and last 5 cue-distractor delays, separately. For the first 5 cue-distractor delays, the correlation was found significantly null (BF_10_ = 0.18). For the last 5 cue-distractor delays, the correlation was found significantly positive (BF_10_ = 33.81). These results show that reaction times (RT) do not simply increase with increasing time between cue and distractor, suggesting the involvement of more than one process.

**Figure 6**. Mean reaction times binned (by step of 39 ms) according to cue-distractor delay (individual data in grey and group data in black). Regression line for the first 5 and last 5 cue-distractor delays are depicted in red and blue, respectively. The corresponding BF_10_ for the Kendall’s Tau correlation are indicated.

**R1.2b. Moreover, the behavioral interaction for RT data is hard to interpret and the authors should do justice in this matter. Since older adults were slower overall, any additional increase in one condition, leading to an interaction, is hard to interpret. The statistical model assumes linearity, but RTs effects may not be linear, but multiplicative. This is a common problem for aging data. The authors want to discuss this.**

We do understand the reviewer’s concern regarding the behavioral interaction reported in our data i.e. increase of reaction times for elderly participants in the late-distractor window. First, we would like to point out that that no significant main effect of age was found. Second, in order to eliminate the non-linearity concerns, for each participant we have normalized reaction times (in each condition) with reaction times in all conditions. Then we tested the influence of (1) age group (2 levels: young and elderly), (2) cue condition (2 levels: informative and uninformative), and (3) distractor condition (3 levels: NoDis, DIS1 and DIS2) on these normalized reaction times using a linear mixed-effects models. Similar to the results reported in section (3.1.2), we found significant main effects of cue category (p = 0.03) and distractor condition (p < 0.01), but the main effect of age was not significant (p = 0.83). In addition, we found a significant interaction between age and distractor factors (p < 0.01). Post-hoc tests indicated that elderly participants were slower than the young ones in the late DIS2 condition (p < 0.01), only. Therefore, when taking account potential global differences in RT, we still find a specific effect of age on RT following late distracting sounds. This effect is unlikely to be due to any ceiling or floor effect.

**Figure 7.** Normalized reaction times in each group according to cue information and distractor conditions. Error bars represent SEM.

**Top-down vs. bottom up:**

**R1.3a. I do not think the interpretation of bottom-up processes is clear here. Larger bottom-up effects should manifest as a larger sensory response to distractors in older compare to younger people. The authors could analyze ERPs to distractor for this. The studies referred to in the discussion (73, 74) show this, but the authors do not discuss these aspects about those studies. The authors also want to consider that sounds were played, on average, at a more intense level to the older compared to younger adults, potentially affecting distraction effects. Note that at suprathreshold sound-levels, sounds may be perceived as louder by older compared to younger people despite hearing loss and the adjustment of sound levels relative to sensation level, due to loudness recruitment/hyperacusis (e.g., changes in cochlear non-linearity and hyperexcitability/ loss of inhibition in auditory cortex). The references 73-75 used to discuss bottom-up effects, may actually reflect top-down processes. In these studies distractors occurred with 50% probability, and subjects may thus prepare for distractors. The data may reflect a preparatory distractor effect, which is not bottom-up. In line 480-, the authors briefly put their “bottom-up” data in the context of reduced top-down inhibition, which obviously is then not bottom-up any longer.**

We thank the reviewer for the opportunity to clarify and to put our results in a more suitable context. We completely agree that in the effects observed in references 73-75 can be explained by top-down inhibitory mechanisms in anticipation of upcoming distracting sounds. It is actually exactly what we meant and the conclusion we made. We do think that the enhanced distractibility observed in elderly is not due to an hyperactivation of the BU attention system but rather to a reduced efficiency of the TD inhibitory system. We have clarified this point in the discussion.

Concerning the intensity level of the distracting sounds, we agree with the reviewer that participants with higher hearing threshold might have perceived the distracting sounds as louder. However, the behavioral measure of distraction is based on the comparison of two conditions with distracting sounds at different positions in time, but with same intensity levels. Also, it is worth mentioning that the ratio between the intensity levels of the distracting and targets sounds is constant across conditions and participants. Therefore, we believe that potential differences in perceived loudness should not account for behavioral differences in distraction effect between groups.

**Figure 8.** mean dB values for target and distracting sounds for each group. Error bars represent standard error of mean.

**R1.3b Furthermore, the authors suggest that top-down processes in for of alpha activity take place in preparation for the sound. Yet, the authors have not shown that the alpha power time courses in their study reflects top-down processes. A contrast between uninformative compared to informative cues could potentially show this. More general in this matter. The authors may want to stay closer to the data. The distinction into top-down and bottom-up is not clear at all here, the concepts are generally under-defined, and are open to interpretation. Staying closer to the data is more appropriate and I recommend the authors do so.**

The role of alpha activity in top-down attention during cueing tasks with long cue-target delays (> 600 ms) has been established in several studies in the somatosensory (Haegens et al. 2014), the visual (Sauseng et al. 2005), and the auditory (Muller and Weisz 2012; Weisz et al. 2014, ElShafei et al. 2018) modalities. Moreover, alpha activity in the auditory cortices following a visual cue and preceding an auditory target can hardly be of bottom-up origin. We have established such a link between alpha in the auditory cortices and top-down attention in a previous study in young adults using a similar paradigm with a more favorable ratio to highlight cue effects (33% left, 33% right, 33% uninformative vs. 25% left, 25% right, 50% uninformative in the present study) (ElShafei et al. 2018, eNeuro). We demonstrated that within the right auditory cortex, alpha power (a) decreased when anticipating a contralateral sound in the low alpha, and (b) increased when anticipating an ipsilateral target sound in the high alpha.

In compliance with the reviewer comment and with reviewer #2 comment R2.3, we further split our virtual regions of interest into left and right hemispheres and analyzed the impact of the cue (Informative vs. Uninformative) on alpha activity. A shown on the figure 9 below, we found a cue effect (reduced alpha power after informative compared to uninformative cues) in the auditory and motor ROIs in the young participants and in the auditory, motor and visual ROIs in the elderly participants. These results provide evidence that alpha activity in our paradigm does reflect top-down processes. This analysis has been added to the manuscript.

**Figure 9**. Mean alpha power (relative to baseline) centered around alpha-peak frequency for each region and each participant, averaged between 600 and 1000 ms (post-cue onset) for each cue condition. **P* < 0.05, **P < 0.01, ***P < 0.001. Error bars represent SEM.

**Linear-mixed effects models:**

**R1.4. The authors need to describe and motivate explicitly which and why (or why not) factors included a random intercept and slope. Choices in this matter can drastically influence the statistical results. In line 248-, for example, I think using fixed effects is inappropriate, since the authors may want to generalize to the population for their age effects.**

We agree with the reviewer and we have now systematically included random intercept and slope for the tested factors when using linear-mixed effects models. To confirm the need for mixed nested models, we have used a likelihood ratio analysis to test the model fit before and after sequential addition of random effects. We used the Akaike Information Criterion and the Bayesian Information Criterion as estimators of the quality of the statistical models generated (Matuschek, Kliegl, Vasishth, Baayen, & Bates, 2017). This has been specified in the method section.

For each analysis, the best model is now indicated in the result section, the corresponding results have been updated. Please note that we had to adjust only one model for reaction time analysis and the results did not change:

Reaction times

- best model: distractor as random slope and subject as random intercept
- DIS effect p < 0.001
- Interaction GROUP by DIS p = 0.036
  - Post-HOC: D2 (OLD > YOUNG; p = 0.069)

**R1.5. Title: “Not Just A Number: Age-Related Modulations of Oscillatory Patterns Underlying Top-Down and Bottom-Up Attention”. I have no idea what “Not Just A Number” refers to. Please remove. Moreover, I recommend that the authors change the title to reflect more what they actually measured instead of “Top-down” and “Bottom-up”, e.g., alpha and gamma activity.**

We thank the reviewer for their recommendation. We have accordingly changed the title into: Age-Related Modulations of alpha and gamma brain activities Underlying Anticipation and Distraction.

**R1.6. Line 69: “impaired gamma response”, I recommend changing it to “altered gamma response”. We do not know what reflects impairment.**

The change has been done.

**R1.7. Line 92: I recommend the authors mention right away that the cue indicates from which location a sound will be played. It was only later that this became clear.**

We thank the reviewer for their suggestion. We have changed the wording accordingly.

**R1.8. Line 197: I recommend that the authors provide more information about the filters they used.**

We have now stated in the methods section that all filtering has been done using Butterworth filters of the third order.

**R1.9. Line 234: The authors need to describe what kind of baseline correction was used.**

We apologize for this omission. We have now clarified that alpha power has been computed relative to a 0.6 to 0.2s pre-cue window.

**R1.10. The alpha frequency analysis felt fairly unmotivated. The authors could improve this.**

We apologize if this section felt unmotivated. Necessary changes in the introduction have been made.

**R1.11. Line 253: It was not fully clear what the authors mean by creating surrogate distractors.**

We apologize for the lack of clarity. We have rephrased the sentence to elucidate the purpose of the surrogate distractors.

**R1.12. The t-values in Figure 7 are very low. How can they be significant? They do not seem very meaningful. Please clarify.**

We agree with the reviewer that the t-values seem fairly low. This is due to massive averaging across time, frequency, or sensors in relatively large space-time-frequency windows.

**R1.13. The authors may also want to cite Zanto et al. 2011 J Neurosci**

We apologize for this omission and we have now added this citation.

**Reviewer #2**: **The authors measured MEG as 14 older and 14 younger adults performed the Competitive Attention Task. Performance did not differ between older and younger groups, but reaction times were slower for older adults, especially in the condition where the distractor was presented later after cue onset. Voxel time series analysis in sensory ROIs showed that older adults have lower alpha power in visual cortex before the onset of the target sound, perhaps suggesting reduced inhibition in task-irrelevant sensory areas. Larger alpha decreases in older adults in motor cortex during the task also suggest a stronger engagement of motor systems to make a response. Gamma oscillations in left dorsolateral prefrontal cortex, left post-central gryus, and left SMA were weaker in older adults. The authors interpret the findings to suggest that older adults have higher distractibility due to modifications of the top-down attention system.**

**I appreciate that the authors investigated an important question regarding the effects of aging on attention. Despite these findings, there are at present several major issues with the conceptualization of the study, the analysis of the results, and the interpretation. These issues will need to be fully addressed before publication is recommended.**

**Data analysis:**

**R2.1. There are several issues with the data processing pipeline that should be addressed. First, it is not clear which type of trials (informative/uninformative/DIS1/DIS2/noDIS) were analysed in the electrophysiological analysis. It appears as if all trials regardless of condition were averaged. This could lead to difficulty in interpretation. I would expect, depending on condition (cued vs uncued), that there would be different time series of alpha in the sensory ROIs. Group differences that depend on condition are obscured and could be skewing the interpretation of the results (e.g., the visual alpha effect in older adults may be due to one condition only). Can the authors provide more details? If the aim is to present the results simply, then only one condition could be analysed (e.g., the condition with the most trials). Further, what were the hemispheric differences of the ROI analysis? This was not shown in the figures. Based on past literature, one might expect that the auditory alpha effect depends on the cued side.**

We apologize for this lack of clarity. For all alpha analyses, we have conducted our analyses using all of the trials with no distractors (NoDIS). For gamma analyses, DIS1 and DIS2 conditions were combined, this dissociation being meaningful for behavioral analysis only. This is now specified in the manuscript.

We do agree with the reviewer’s concern that group differences could be condition dependent.
To address this concern, we have investigated the impact of the cue information on both alpha and gamma activities.

[1] As mentioned in response to reviewer #1 comment (R1.3b), we investigated the impact of the cue (Informative vs. Uninformative) on alpha activity (at the individual peak frequency) at the ROI level. A linear mixed-effects model was fit to predict modulation of alpha activity between 600 and 1000ms (relative to cue onset) with the following factors: (1) group (old, young), (2) modality (auditory, visual and motor), (3) hemisphere (left and right) and (4) cue condition (informative and uninformative). This analysis also addresses the question of inter-hemispheric differences. As shown on the figure 8, we found a cue effect (reduced alpha power after informative compared to uninformative cues) in the auditory and motor ROIs in the young participants and in the auditory, motor and visual ROIs in the elderly participants. We also found for all cue conditions that the alpha power in visual ROIs was significantly larger in the younger group than in elderly (main effect of group p<0.001).

[2] As mentioned in section ONE, we have run an additional analysis to investigate the impact of the cue information on gamma power in each group. Young participants presented an increase in gamma power in median frontal regions for informative cue compared to uninformative cue, while no significant difference was found in elderly.

These analyses have been added to the manuscript.

**R2.2. Another issue with the data analysis is the unstructured manner in which the alpha effects and gamma effects were conducted. The gamma analysis arose from a data-driven approach where distributions of t-values in sensor space helped define time and frequency ROIs for the DICS beamformer. The same approach was not given to alpha activity, which was done by simply choosing sensory ROIs. This method bypasses possibly important information about top-down alpha activity in non-sensory areas that could be different between younger and older individuals. I would recommend that the authors use a like-minded data-driven approach to compute time series of alpha activity for all voxels across the brain, and perhaps restrict them to all Brodmann areas to reduce the data space. The cluster-based analysis could be re-analyzed, this time also correcting across spatial location.**

The alpha analysis was based on strong hypotheses regarding regions of interest (ROIs: visual and auditory) and frequency bands (low and high) where alpha activity would be modulated during our task. These hypotheses are based upon a previous study using a similar paradigm in a different group of young participants (ElShafei et al., 2018). Nevertheless, we have addressed the reviewer concern at the beginning of this document in a common response to both reviewers (section ONE). We have indeed used a data-driven approach with the DICS beamformer and Cluster Based Permutation Tests to analyze alpha activities.

**R2.3. The peak alpha analysis also raises concerns on the alpha time series analysis. Clearly there are large individual differences in peak alpha. The qualitative differences in alpha in motor ROI between young and older individuals may account for the effect that was found in high alpha in the time series analysis. What would happen to the time series analysis if it was analyzed at the peak frequency of each participant? Would effects remain? The authors recognize this (l. 444 to 448) and have the opportunity to address it, but do not.**

We thank the reviewer for raising such an important concern. We do agree that analyzing the time-series around each participant’s peak frequency is a much more suitable approach. As seen in Figure 10, this analysis does not change our effect i.e. we still observe a more prominent alpha increase in visual areas for the younger group, with a more prominent alpha decrease in motor areas for the older group. This analysis has been included in the manuscript.

**Figure10**: Time-course of alpha activity centered around alpha-peak frequency for each region and each participant.

**R2.4. The authors use linear mixed effects models (cited in l 166 and l 249) and reported interactions. However there appeared to be no term for random slope in their model, which leads to higher type I error rates. The authors should repeat the analysis including a term for random slope over the within-subjects factors.**

We agree with the reviewer and we have now systematically included random intercept and slope for the tested factors when using linear-mixed effects models. To confirm the need for mixed nested models, we have used a likelihood ratio analysis to test the model fit before and after sequential addition of random effects. We used the Akaike Information Criterion and the Bayesian Information Criterion as estimators of the quality of the statistical models generated (Matuschek, Kliegl, Vasishth, Baayen, & Bates, 2017). This has been specified in the method section. For each analysis, the best model is now indicated in the result section, the corresponding results have been updated. Please note that, though we had to adjust our models, the results did not change for reaction times, % of incorrect responses and for the alpha peak frequency.

**R2.5. Finally, on l. 85, the authors should describe which data of younger individuals was analyzed in a previous report. The issue of "double dipping" data "or circular analysis is a concern when re-using past data, which can lead to higher false alarm rates if a correction factor is not applied.**

We thank the reviewer for the opportunity to clarify this point. In another (now published ElShafei et al, Cerebral Cortex, 2019) study, we have investigated the brain networks activated in response to distracting sounds in a cohort of 21 young (<30 year-old) participants out of which 14 have been chosen as matching young controls to the older participants in the present study. We respectfully disagree that our analysis is confounded by double dipping as the two studies tackled two different research questions.

**Introduction and conceptualization of attention and oscillations:**

**R2.6. The characterization of attention consisting of top-down and bottom-up processes is a bit oversimplified. Whereas this conceptualization has enjoyed mainstream focus because it is parsimonious, there are some issues with this view (see Awh et al. 2012; Trends Cogn Sci. 2012 Aug; 16(8): 437–443) such that forms of attention are better described by task goals. For instance, "bottom-up" capture (and its neural mechanisms) can be entirely changed depending on the attentional (task) demands placed on the observer. In the present task, bottom-up effects are never fairly assessed because the attentional resources are directed to the cued sound discrimination task---the distractor was never more than a "check" on the listener's top-down ability to focus categorizing the pitch. If a design were to also include a task-switching component where an unexpected, salient stimulus became the object of focus, then bottom-up systems might be more accurately analyzed. In a similar vein, the conceptualization of oscillations as fast and slow respectively relating to top-down and bottom-up systems is oversimplified. Alpha oscillations, under the "functional inhibition" hypothesis, do appear to serve a suppressive role in either inhibiting task-irrelevant brain areas or stimuli that are to-be-ignored. However, depending on task and brain region, alpha (power and phase) may also have a positive relationship with neural excitation (Palva and Palva, 2007; Trends in Neurosciences, Vol 30 No 4). Similarly, Gamma oscillations are similarly complex, and the authors also acknowledge that gamma may have a top-down inhibitory role in lines 469-473. In short it is a bit disingenuous to the breadth of research to simply refer to only top-down and bottom-up mechanisms, and their respective relation to slow and fast oscillations. This may apply to limited circumstances in some research paradigms, but they are not a generalization, and not universally accepted. Importantly, these conceptualizations are not presently supported by the data presented here. I recommend that the authors should re-cast the introduction and discussion so that a balanced perspective is given to the reader.**

We thank the reviewer for their comment. Our aim was certainly not to bypass the breadth of the existing. The introduction and discussion sections have now been edited to highlight the exact processes that we investigate.

**R2.7. l. 77 - Please provide additional details on how scholarship and musical education were quantified and how data were collected.**

Data regarding scholarship and musical education were collected by asking the participants the latest educational degree obtained and the number of years of formal musical training, respectively.

**R2.8. Section 2.2.1 - I found this section more difficult to follow than it probably was. I recommend starting by describing the trial structure more generally and then referring to the 75% and 25% conditions once the reader grasps the general flow of stimulus presentation.**

We apologize for the unwanted density of this section. We have now changed the phrasing of this section.

**R2.9. Section 2.2.2 - Can the authors expand on their choice of tone discrimination? I understand that all participants were able to discriminate the tones, but the closeness of pitch between "high" and "low" tones may be introducing a memory component. For instance, if the authors chose 450 Hz vs 1100 Hz, which ought to still be in the normal hearing region for most participants, the "high" and "low" become easily marked categories. But with pitches close together, they may have to rely on memory of the relative differences established during the initial discrimination task. Such memory demands could impinge on results. During the actual study, what were reaction times and performance measures like for individuals who had increased pitch size differences due to detection problems?**

We thank the reviewer for the opportunity to clarify. We do agree that tone discrimination in our task could be conceived as difficult. However, participants in both groups performed the task quite well with no differences between the groups. As suggested by the reviewer, we have separated participants according to the pitch difference, namely “standard” (two semitones of difference, n=15) and “adapted” (difference between 2.5 and 3 semitones, n=13). Paired t-tests revealed no significant differences neither median for reaction times (p = 0.078) nor in percentage of incorrect responses (p=0.44). There seems to be no major difference in performances between individuals with increased pitch difference and the others.

**Figure 11.** Median reaction time and percentage of incorrect responses for participants according to the pitch difference used during the experiment. Within each boxplot (Tukey method), the horizontal line represents the median, the box delineates the area between the first and third quartiles (interquartile range).

**R2.10. l. 174-175: The concept of "arousal benefit" and "attention capture" are not clear with respect to the condition subtractions. How and why do the authors feel that these contrasts represent arousal and attention, and what evidence supports this?**

We apologize for the lack of justification for these contrasts. Please refer to our response to comment (R1.2a) for a detailed explanation.

**R2.11a. Figure 2: Has a typo in the word "Distractor"**

**R2.11b. l. 223 - "beamformer" duplicated in this sentence**

**R2.11c. l. 400 - "You" appears erroneously at the beginning of this sentence**

**R2.11d. l. 13 - "For" appears erroneously at the beginning of this sentence.**

Thank you for pointing these typos out. All of them have been corrected.

**R2.12. l. 258 - The use and description of the "surrogate distractor" is not clear and muddies the understanding of the analysis of gamma activity. Please re-word.**

We apologize for the lack of clarity. We have rephrased the sentence to elucidate the purpose of the surrogate distractors.

**R2.13. l. 405 - Please describe what is meant by "recruitment" here.**

We apologize for confusion. We have used the term recruitment as a synonym for activation, this has been modified.

**R2.14. l. 444 - Formally (not colloquially) this is not the correct usage of "begging" the question. The authors probably intend to say "raise the question"**

We have changed the sentence upon the reviewer’s suggestion.
