# Supplementary material for: Age-related modulations of alpha and gamma brain activities underlying anticipation and distraction
Source: PLoS One. 2020 Mar 12;15(3):e0229334. doi: 10.1371/journal.pone.0229334 (PMC7067396; doi:10.1371/journal.pone.0229334)
Supplement: Attachment — Submitted filename: elshafei et al second round Response to Reviewers.docx [file pone.0229334.s007.docx]

We thank the reviewers for their positive feedback on our revised manuscript. We truly believe that, based upon their comments, the manuscript has greatly improved. We hope that this revised version would address all of the reviewers’ concerns. Below, we have laid out the reviewers’ comments (in blue font) along with our replies in black. We have taken the liberty to number the reviewer’s comments so that they are easier to refer to. In the revised manuscript, modifications are highlighted in yellow.

Reviewer #1: Revision - Age-Related Modulations of Alpha and Gamma Brain Activities Underlying Anticipation and Distraction

I thank the authors for their efforts to respond to my comments. The manuscript has improved substantially. I have still a few comments

Major:

Data visualization and corresponding text:

1. The images and plots are small in some instances and make it hard to identify what the authors describe in the text. For example, Figure 5 includes lots of unnecessary white space and the TFR plots are small. It may also be worth considering to reduce the frequency range plotted in the TFR plots to 20 Hz or 25 Hz as the upper limit. The authors do not report any effects above 15 Hz. The large range makes it difficult to see the relevant details. It was also not clear whether the authors conducted a direction comparison between age groups for the data displayed in Figure 5. It was also not clear why a p<0.1 threshold was chosen for this figure (0.05 in others). The plots in Figure 6 and 10 could also be made bigger and a color bar could be chosen that allows identifying better where the peak maxima are in the maps. T-values displayed in Figure 9 are small. Are they indeed significant? (z-maps may be better in the future because they are independent of the DFs). Figure 10 and corresponding text: Was the cue by age group interaction significant? It was not reported. The authors are careful in their wording, which is good. Nevertheless, they may want to be explicit (Page 32: The authors may also explicitly state that the interaction was not significant, if so, to avoid implicit implications).

We apologize for these inaccuracies. We have revised all of our figures, increased font size and reduced white space.

- For figure 5: we have reduced the frequency range to 25Hz. Only, for that figure, a p<0.1 threshold was chosen to elucidate a trending effect of great relevance i.e. distinct (low/high) alpha sub-bands support distinct (facilitatory/inhibitory) top-down attentional mechanisms, in the younger group.
- For figure 6 and 10, we have adapted the color bars to highlight the peak maxima. We also changed the text on the figures and the captions since no direction comparison between groups has been performed.
- We agree with the reviewer that the t-values in Figure 6 seem fairly low. This is due to (1) averaging across time, frequency, or sensors in relatively large space-time-frequency windows, and (2) relative low signal-to-noise ratio of high frequency activity. However, these values are indeed significant as indicated by the p-value of the cluster (p < 0.001).
- In figure 10, we do not directly test for a cue by group interaction, due to the limitations of cluster-based permutation tests. Thus, we perform the similar test (informative versus uninformative) for each group separately. A note has been added to the manuscript to clarify this point.

Minor:

2. Page 12: The authors write “… Informative NoDIS and of distractor effects on the differences in RTs NoDIS – DIS1 (as a measure of the AROUSAL BENEFIT) or DIS2 – DIS1 (as a measure of DISTRACTION COST) …” It is not explained nor clear why “Arousal Benefit” and “Distraction Cost” would be associated with the different distraction time window. I recommend removing this, since the authors do not come back to it at other places in the manuscript.

We respectfully disagree with the reviewer, but we do come back to that distinction in the Results (section 3.2.1) and the Discussion (section 4.1) for which we now provide a schematic representation in Figure1 below. Thus, we prefer not to remove that sentence. In addition, the justification for using these differences can be found in the cited references:

[1] Bidet-Caulet A, Bottemanne L, Fonteneau C, Giard M-H, Bertrand O. Brain Dynamics of Distractibility: Interaction Between Top-Down and Bottom-Up Mechanisms of Auditory Attention. Brain Topogr. 2014;28(3):423–36.

[2] Masson R, Bidet-Caulet A. Fronto-central P3a to distracting sounds: An index of their arousing properties. NeuroImage. 2019 Jan 15;185:164–80.

**Figure1.** Schematic representation of the behavioral effects of distracting sounds in the Competitive Attention Test.

3. Page 14: The authors provide a bit more information about source localization. Some of the information is not fully clear though. Maybe the authors could clearly identify what they used as a volume conductor and what as source model. The two seem somewhat entangled currently. The authors may also want to comment on how they morphed the MNI template was morphed to each individual brain.

We apologize for the lack all of clarity. We now report additional information (e.g. tapers, regularization factor) for our source localization methods. In addition, we clearly state that:

*“For each participant, an anatomically realistic single-shell headmodel (volume conduction model) based on the cortical surface was generated from individual head shape. A grid (source model) with 0.5-cm resolution was created using an MNI template, and then morphed (adjusted) into the brain volume of each participant using non-linear transformation. This procedure ensures that each grid-point would represent the same anatomical label across all subjects but with different subject-specific head positions.”*

4. Data availability: The data are not made available at this point, but the authors mention that the data are available upon request. This may be sufficient for PLoS ONE, I only wanted to raise the issue, because it was one of the points in the drop-down menu.

We thank the reviewers for raising this point. This point has been addressed directly with the editor.

Reviewer #2: I thank the authors for addressing my concerns. The changes the authors have made have been clearly outlined, and I also thank the authors for their additional efforts to articulate the comments from both reviewers, providing supplemental flow charts and plots supporting their responses. My opinion is that the quality of the manuscript has been greatly improved. Below I have additional comments based on the new results and changes.

1. With the results now fully understood, there is an outstanding concern with the stimulus design that impacts the interpretation of the results. Stimulus levels for both target and distractor sounds were set based upon the threshold for the target stimuli. The target stimuli were between 512 to three semitones above 575 Hz. However, the distractor stimuli were complex sounds (phone rings, alarms, etc) which likely have acoustic spectra that reach much higher frequencies (if the authors could supply those, this would be helpful). Older adults commonly have elevated thresholds at frequencies higher than the target stimulus. Thus, the sensation level of the distractor tone, which was based upon the threshold of the target tone, was most likely lower for older adults than it was for younger adults. No audiograms were supplied (or apparently collected) which could indicate otherwise. If older adults received less salient (i.e., loud) distractor stimuli than younger adults, the difference has consequences for the interpretation of the "bottom-up" effect of the distractor as a function of age. Older adults had evidence of reduced activation of gamma activity in lateral prefrontal regions compared to younger adults. Because of varying sensation levels in older adults, it cannot be determined if this effect is due to aging or lower sensation level of the distractor due to hearing loss. At minimum, this is a major caveat that should condition the interpretation provided by the authors. While there does seem to be evidence of reduced top-down "filtering" via weaker modulation of visual alpha, the role of the "bottom-up" attention network remains equivocal. The conclusions of the study (e.g., entire section 4.3, 4.4, opening of section 4) likely need to be adjusted.

This is a very relevant remark. As shown in Figure2, the acoustic spectra for some of the distracting sounds reach high frequencies while some are restricted to rather low frequencies (below 1500 Hz).

As shown in Figure3, we split the distracting sounds into 18 low and 18 high frequency sounds (using 1500Hz as the border) and re-run the behavioral analysis on median reaction times (general linear mixed models) with three factors (age, distracting-sound delay and distracting-sound frequency). Similarly to our original analysis, we found a main effect of distracting-sound delay (F(2,27) = 118.39, p < 0.001, η^2^ = 0.53) on reaction times and a significant interaction between age and distractor sound delay (F(1,27) = 16.95, p < 0.001, η^2^ = 0.17). However, we found neither a significant main effect of significant distractor sound frequency (F(2,27) = 0.13, p = 0.71, η^2^ = 0.002), nor a significant interaction between age and distractor sound frequency (F(1,27) = 1.93, p = 0.16, η^2^ = 0.024).

These results were confirmed by a Bayesian repeated-measure ANOVA with the same factors. This analysis showed that the best model accounting for the data is the one with the main effect of distracting-sound delay and the interaction between age and distracting-sound delay (BF_10_ = 4.1e+14). There was decisive evidence for the effect of distracting-sound delay (BF_inclusion_=9.5e +13) and the interaction between age and distracting-sound delay (BF_inclusion_=232.4), strong evidence for the effect of age (BF_inclusion_=77.2), no evidence for the interaction between distracting-sound frequency and position (BF_inclusion_=1.0), the interaction between age and distractor sound frequency (BF_inclusion_=0.6), and the triple interaction (BF_inclusion_=0.6), and positive evidence against the effect of distracting-sound frequency (BF_inclusion_=0.3).

These two analysis strongly suggest that, irrespective of the frequency content of distracting sounds, elderly participants show longer reaction times after late distracting sounds. In other words, elderly show the same enhanced distraction effect irrespective of the frequency content of distracting sounds. Therefore, it seems quite unlikely that results observed at the brain level are due to differences in sensation level between elderly and young participants.

**Figure2.** Acoustic spectra for the 40 distracting sounds used for the experiment.

**Figure3.** Median reaction times in each group according to distracting-sound delay and frequency conditions.

2. As a brief comment, was there an attempt to predict inter-individual behavioral performance from the neural variables? This might strengthen the arguments for or against the balance of top-down filtering via alpha or modulation of distractor processing in attention networks via gamma.

This is a very good suggestion. We have indeed computed correlations between behavioral performances (distraction effect and cue benefit) and neural variables (alpha and gamma activity). However, no test reached statistical significance when performed on a whole brain level i.e. with no pre-defined regions of interest. However, upon defining four regions of interest (lateral prefrontal cortex, visual, auditory and motor cortices), we have found significant correlations only between the distraction effect and:

- Gamma activity in the lateral prefrontal cortex and (r_spearman_= - 0.37, p = 0.04).
- Alpha activity (0.6-1 s, post-cue) in the visual cortex, i.e. marker of top-down suppression (r_spearman_= - 0.43, p = 0.02).

In addition, the aforementioned activities were also significantly correlated (r_spearman_= 0.41, p = 0.02). Interestingly, the distraction effect did not correlate with alpha activity in the auditory nor motor cortex, i.e. markers of top-down facilitation.

**Figure4**. Scatter plots of behavioral distraction effect against alpha activity in the visual cortex and gamma activity in the lateral prefrontal cortex.

3. It remains unclear to me how the DIS1 and DIS2 time windows were determined. Based on a reply to the other reviewer, the authors establish that there is not a linear increase of RT as a function of the distractor time. However the binning of RT by 39 ms steps (instead of plotting a continuous function) obscures any obvious pivot points as a function of time that suggest that later distractors are of a separate process. In other words, Figure 6 is not convincing as to support their DIS1/2 windows. Bins 3,4,and 5 on the abscissa could conceivably belong to either the early or the late windows. This has implications for both the analysis windows for neural variables and also behavior, and it would benefit the paper if there was a stronger motivation.

We apologize if the choice for distractor delays remains unclear. The binning was done in such a way to encompass sufficient trials since trials with distractors constitute only 25% of trials. Thus, plotting the data in a continuous manner would not be informative and rather noisy.

As shown in Figure1 (above), the dissociation between DIS1 and DIS2 time-windows is only relevant for behavior analysis, since the impact of the two phenomena triggered by a distracting sound (arousal burst and distraction) on the reaction times to target differ according to the distractor-target delay. This dissociation provides a good approximation at the behavioral level of the strength of the arousal burst and distraction phenomena. At the brain level, this dissociation would be rather meaningless, since both phenomena are triggered irrespective of the distractor-target delay. Therefore, the brain analyses have been performed on the average of all distracting sounds.

4. This is more of a stylistic suggestion---axis labels and text in some of the figures could be enlarged. For instance, Figures 5 and 9 have very small text for axis ticks, but other figures (behavioral ones) enjoy larger text.

We thank the reviewer for this suggestion. We have revised all of our figures, increased font size and reduced white space

5. As a final comment, it would be helpful for the manuscript if the authors could go through carefully and adjust the very minor grammatical and style errors that remain throughout the manuscript. Some examples:

l. 43: there should be a hyphen, not an n-dash, between "top" and "down"

l. 87-88 ranges should be separated by n-dashes, not m-dashes.

l. 110: no hyphen is needed between "grey" and "background.

l. 112: "only" is not needed in this sentence.

l. 113-115: after "DIS2," use the word "and" or use semicolons to properly break up the clauses in this sentence

l. 120: "the" before "distracting sound"

l. 135: "the" before "informative cue"

This list is not exhaustive.

We apologize for these mistakes. We have now fully proofread the manuscript for such errors.
