# Supplementary material for: Age-related modulations of alpha and gamma brain activities underlying anticipation and distraction
Source: PLoS One. 2020 Mar 12;15(3):e0229334. doi: 10.1371/journal.pone.0229334 (PMC7067396; doi:10.1371/journal.pone.0229334)
Supplement: Attachment — Submitted filename: elshafei et al third round Response to Reviewers.docx [file pone.0229334.s008.docx]

We thank both reviewers for their positive feedback on our revised manuscript. Below, we have laid out the reviewers’ remaining comments (in blue font) along with our reply in black. In the revised manuscript, modifications are highlighted in yellow.

Reviewer #2:

I thank the authors for addressing my comments and providing visualization for an explanation. They have been clear and concise in the response. I have only one remaining concern:

The authors wrote: "These two analysis strongly suggest that, irrespective of the frequency content of distracting sounds, elderly participants show longer reaction times after late distracting sounds". The above comment in reference to a potential confound with sensation level for distracting sounds for the older adults.

I appreciate the detailed analysis showing that the stimuli with acoustically higher frequencies did not have an observable effect on behavior. However my comment originally was concerned with the effect on gamma activity, which was not addressed in the authors' response. In a separate comment the authors found that gamma activity was only weakly correlated with the behavioral distractor effect, accounting for very little of the variance. Thus the absence of an effect on behavioral data does not preclude an effect on the neural data, as well as the interpretation of this effect. Unless otherwise supported by data, the caveat warrants a note of limitation in the discussion of gamma results in older adults, and the discussion of the results in section 4.3 should be tempered.

We understand the reviewer’s concern. Accordingly, we have addressed such limitation in the manuscript:

“*It is important to note that, in the present study, distracting sounds were complex sounds with acoustic spectra reaching high frequencies where older participants can display altered sensation levels. These potential differences in sensation levels between groups could result in neural activity differences. However, we believe that differences in sensation levels, related to peripheral damage, would have mostly manifested as differences in gamma activation in the ventral BU attentional network, in particular in the auditory cortices, rather than in lateral prefrontal regions. Due to the relatively low number of trials with distracting sounds and low signal-to-noise ratio of gamma activity, we were not able to factor spectral content of distracting sounds in our gamma analyses. However, the behavioral analysis on median reaction times (supplementary figure 5) according to the low or high spectral content of the distracting sounds showed neither a significant main effect of spectral content, nor a significant interaction between age and spectral content. While the absence of an effect on behavioral data does not preclude an effect on the neural data, we believe that potential differences in sensation levels is quite unlikely to explain differences in gamma activation between groups.”*
