# Supplementary material for: Age-related modulations of alpha and gamma brain activities underlying anticipation and distraction
Source: PLoS One. 2020 Mar 12;15(3):e0229334. doi: 10.1371/journal.pone.0229334 (PMC7067396; doi:10.1371/journal.pone.0229334)
Supplement: Peer review history

# Peer review: Decision Letter 0

**DOI:** 10.1371/journal.pone.0229334.r001

**Authors:** Claude Alain

6 Sep 2019

PONE-D-19-15009

Not Just A Number: Age-Related Modulations of Oscillatory Patterns Underlying Top-Down and Bottom-Up Attention.

PLOS ONE

Dear Dr. ElShafei,

Thank you for submitting your manuscript to PLOS ONE. After careful consideration, we feel that it has merit but does not fully meet PLOS ONE’s publication criteria as it currently stands. Therefore, we invite you to submit a revised version of the manuscript that addresses the points raised during the review process.

We would appreciate receiving your revised manuscript by Oct 21 2019 11:59PM. When you are ready to submit your revision, log on to https://www.editorialmanager.com/pone/ and select the 'Submissions Needing Revision' folder to locate your manuscript file.

If you would like to make changes to your financial disclosure, please include your updated statement in your cover letter.

To enhance the reproducibility of your results, we recommend that if applicable you deposit your laboratory protocols in protocols.io, where a protocol can be assigned its own identifier (DOI) such that it can be cited independently in the future. For instructions see: http://journals.plos.org/plosone/s/submission-guidelines#loc-laboratory-protocols

Please include the following items when submitting your revised manuscript:

- A rebuttal letter that responds to each point raised by the academic editor and reviewer(s). This letter should be uploaded as separate file and labeled 'Response to Reviewers'.
- A marked-up copy of your manuscript that highlights changes made to the original version. This file should be uploaded as separate file and labeled 'Revised Manuscript with Track Changes'.
- An unmarked version of your revised paper without tracked changes. This file should be uploaded as separate file and labeled 'Manuscript'.

Please note while forming your response, if your article is accepted, you may have the opportunity to make the peer review history publicly available. The record will include editor decision letters (with reviews) and your responses to reviewer comments. If eligible, we will contact you to opt in or out.

We look forward to receiving your revised manuscript.

Kind regards,

Claude Alain

Academic Editor

PLOS ONE

Journal Requirements:

When submitting your revision, we need you to address these additional requirements.

1. Please ensure that your manuscript meets PLOS ONE's style requirements, including those for file naming. The PLOS ONE style templates can be found at http://www.journals.plos.org/plosone/s/file?id=wjVg/PLOSOne_formatting_sample_main_body.pdf and http://www.journals.plos.org/plosone/s/file?id=ba62/PLOSOne_formatting_sample_title_authors_affiliations.pdf

2.  We note that you have indicated that data from this study are available upon request. PLOS only allows data to be available upon request if there are legal or ethical restrictions on sharing data publicly. For more information on unacceptable data access restrictions, please see http://journals.plos.org/plosone/s/data-availability#loc-unacceptable-data-access-restrictions.

In your revised cover letter, please address the following prompts:

a) If there are ethical or legal restrictions on sharing a de-identified data set, please explain them in detail (e.g., data contain potentially sensitive information, data are owned by a third-party organization, etc.) and who has imposed them (e.g., an ethics committee). Please also provide contact information for a data access committee, ethics committee, or other institutional body to which data requests may be sent.

b) If there are no restrictions, please upload the minimal anonymized data set necessary to replicate your study findings as either Supporting Information files or to a stable, public repository and provide us with the relevant URLs, DOIs, or accession numbers. For a list of acceptable repositories, please see http://journals.plos.org/plosone/s/data-availability#loc-recommended-repositories.

We will update your Data Availability statement on your behalf to reflect the information you provide.

Additional Editor Comments (if provided):

[Note: HTML markup is below. Please do not edit.]

Reviewers' comments:

Reviewer's Responses to Questions

**Comments to the Author**

1. Is the manuscript technically sound, and do the data support the conclusions?

The manuscript must describe a technically sound piece of scientific research with data that supports the conclusions. Experiments must have been conducted rigorously, with appropriate controls, replication, and sample sizes. The conclusions must be drawn appropriately based on the data presented. 

Reviewer #1: Partly

Reviewer #2: Partly

**********

2. Has the statistical analysis been performed appropriately and rigorously? 

Reviewer #1: I Don't Know

Reviewer #2: No

**********

3. Have the authors made all data underlying the findings in their manuscript fully available?

The PLOS Data policy requires authors to make all data underlying the findings described in their manuscript fully available without restriction, with rare exception (please refer to the Data Availability Statement in the manuscript PDF file). The data should be provided as part of the manuscript or its supporting information, or deposited to a public repository. For example, in addition to summary statistics, the data points behind means, medians and variance measures should be available. If there are restrictions on publicly sharing data—e.g. participant privacy or use of data from a third party—those must be specified.

Reviewer #1: No

Reviewer #2: No

**********

4. Is the manuscript presented in an intelligible fashion and written in standard English?

PLOS ONE does not copyedit accepted manuscripts, so the language in submitted articles must be clear, correct, and unambiguous. Any typographical or grammatical errors should be corrected at revision, so please note any specific errors here.

Reviewer #1: Yes

Reviewer #2: Yes

**********

5. Review Comments to the Author

Please use the space provided to explain your answers to the questions above. You may also include additional comments for the author, including concerns about dual publication, research ethics, or publication ethics. (Please upload your review as an attachment if it exceeds 20,000 characters)

Reviewer #1: Review - Not Just A Number: Age-Related Modulations of Oscillatory Patterns Underlying Top-Down and Bottom-Up Attention

The authors of this manuscript describe an MEG study that aims to investigate age-related differences in auditory top-down and bottom-up processes. The authors show differences in alpha power between younger and older adults that are interpreted to reflect top-down processes. They also find differences in gamma-band activity between age groups that are interpreted to reflect bottom-up processes. The topic is interesting and the study could be received well by the community. However, I have some concerns regarding the data analysis and interpretation of the data, described in detail below:

Major:

(1) Alpha power analysis:

The alpha-power analysis and data presentation appear selective and not fully straight forward. The authors use a time-domain spatial filter (LCMV) to project data into source/virtual space and then analyze data in the frequency domain. That is, the filter may be based on very different, non-alpha activity, aspects of the data. It is not clear how this affects the results. For the gamma band they use, more appropriately, a spatial filter for frequency data (DICS). It is not clear why two different approaches were chosen, because the DICS beamformer would have been the appropriate choice also for the alpha data. I recommend making the data analysis consistent and using a DICS beamformer throughout. Moreover, despite focusing on a specific frequency band (alpha), the authors still want to show full time frequency plots from the wavelet analysis, similar to what they have done for the gamma band. The reader wants to see how power spreads across frequencies, which also helps to get an idea about differences in alpha frequency. Moreover, full source localizations for the alpha activity should be provided. From the plots in Figure 5, it is not sufficiently clear that there are actually multiple different sources. It looks like there may also be effects of volume conduction present. Furthermore, it was not clear why the alpha band was divided into fairly wide subbands. It is very uncommon to go up to 15 Hz, and this would need to be justified. The differences in alpha frequency may further impact effects of alpha power, using a broad frequency window, because the frequency window chosen may not appropriately reflect power, if there indeed different sources with different best frequencies. In short, the authors want to show time-frequency plots for alpha data, use the DICS beamformer for alpha activity, and show full source localizations for alpha power. This should be no problem, given that the authors used this approach for the gamma band.

Finally, the authors, in several places of the manuscript, mention that for auditory cortex there is an alpha desynchronization whereas for visual cortex there is alpha synchronization. There is no reason, based on what the time courses look like, to assume that there are functional differences. A reduction from baseline present is present in all regions, just that the increase in power happens to become positive in a later time window for the visual region. Some of the differences could come from volume conduction. The authors want to tune down interpretations and wording in this regard (e.g., it reads oddly in line 331ff that power was lower for older compared to younger adults for two contrasts, but that one was referred to as synchronization and one as desynchronization; this has also implications do the interpretation of facilitation and suppression).

(2) Interpretation / methods for behavioral data:

The separation into an early and late time window during which a distractor may occur could be motivated more strongly. In fact, the authors make a clear distinction about the processes that happen for early vs. late distractors. I am not convinced that this is necessarily the case and I also do not understand how they came up with the processes they attribute to the early and late time window. Assuming the distractors were uniformly spaced in time (the authors want to describe more explicitly how distractors were spaced), the data could also be analyzed more continuously, e.g., fitting a linear function to the RT data as a function of distractor time. The choice of separating an early and late time window is a methodological choice that may impact interpretation here. It may actually be that RTs “just” increase with increasing time between cue and distractor. This would suggest one process, not two.

Moreover, the behavioral interaction for RT data is hard to interpret and the authors should do justice in this matter. Since older adults were slower overall, any additional increase in one condition, leading to an interaction, is hard to interpret. The statistical model assumes linearity, but RTs effects may not be linear, but multiplicative. This is a common problem for aging data. The authors want to discuss this.

(3) Top-down vs. bottom up:

I do not think the interpretation of bottom-up processes is clear here. Larger bottom-up effects should manifest as a larger sensory response to distractors in older compare to younger people. The authors could analyze ERPs to distractor for this. The studies referred to in the discussion (73, 74) show this, but the authors do not discuss this aspects about those studies. The authors also want to consider that sounds were played, on average, at a more intense level to the older compared to younger adults, potentially affecting distraction effects. Note that at suprathreshold sound-levels, sounds may be perceived as louder by older compared to younger people despite hearing loss and the adjustment of sound levels relative to sensation level, due to loudness recruitment/hyperacusis (e.g., changes in cochlear non-linearity and hyperexcitability/ loss of inhibition in auditory cortex).

The references 73-75 used to discuss bottom-up effects, may actually reflect top-down processes. In these studies distractors occurred with 50% probability, and subjects may thus prepare for distractors. The data may reflect a preparatory distractor effect, which is not bottom-up. In line 480ff, the authors briefly put their “bottom-up” data in the context of reduced top-down inhibition, which obviously is then not bottom-up any longer.

Furthermore, the authors suggest that top-down processes in for of alpha activity take place in preparation for the sound. Yet, the authors have not shown that the alpha power time courses in their study reflects top-down processes. A contrast between uninformative compared to informative cues could potentially show this.

More general in this matter. The authors may want to stay closer to the data. The distinction into top-down and bottom-up is not clear at all here, the concepts are generally under-defined, and are open to interpretation. Staying closer to the data is more appropriate and I recommend the authors do so.

(4) Linear-mixed effects models:

The authors need to describe and motivate explicitly which and why (or why not) factors included a random intercept and slope. Choices in this matter can drastically influence the statistical results. In line 248ff, for example, I think using fixed effects is inappropriate, since the authors may want to generalize to the population for their age effects.

Minor:

- Title: “Not Just A Number: Age-Related Modulations of Oscillatory Patterns Underlying Top-Down and Bottom-Up Attention”. I have no idea what “Not Just A Number” refers to. Please remove. Moreover, I recommend that the authors change the title to reflect more what they actually measured instead of “Top-down” and “Bottom-up”, e.g., alpha and gamma activity.

- Line 69: “impaired gamma response”, I recommend changing it to “altered gamma response”. We do not know what reflects impairment.

- Line 92: I recommend the authors mention right away that the cue indicates from which location a sound will be played. It was only later that this became clear.

- Line 197ff: I recommend that the authors provide more information about the filters they used.

- Line 234ff: The authors need to describe what kind of baseline correction was used.

- The alpha frequency analysis felt fairly unmotivated. The authors could improve this.

- Line 253: It was not fully clear what the authors mean by creating surrogate distractors.

- The t-values in Figure 7 are very low. How can they be significant? They do not seem very meaningful. Please clarify.

- The authors may also want to cite Zanto et al. 2011 J Neurosci

Reviewer #2: The authors measured MEG as 14 older and 14 younger adults performed the Competitive Attention Task. Performance did not differ between older and younger groups, but reaction times were slower for older adults, especially in the condition where the distractor was presented later after cue onset. Voxel time series analysis in sensory ROIs showed that older adults have lower alpha power in visual cortex before the onset of the target sound, perhaps suggesting reduced inhibition in task-irrelevant sensory areas. Larger alpha decreases in older adults in motor cortex during the task also suggest a stronger engagement of motor systems to make a response. Gamma oscillations in left dorsolateral prefrontal cortex, left post-central gryus, and left SMA were weaker in older adults. The authors interpret the findings to suggest that older adults have higher distractibility due to modifications of the top-down attention system.

I appreciate that the authors investigated an important question regarding the effects of aging on attention. Despite these findings, there are at present several major issues with the conceptualization of the study, the analysis of the results, and the interpretation. These issues will need to be fully addressed before publication is recommended.

MAIN ISSUES

Data analysis:

There are several issues with the data processing pipeline that should be addressed. First, it is not clear which type of trials (informative/uninformative/DIS1/DIS2/noDIS) were analyzed in the electrophysiological analysis. It appears as if all trials regardless of condition were averaged. This could lead to difficulty in interpretation. I would expect, depending on condition (cued vs uncued), that there would be different time series of alpha in the sensory ROIs. Group differences that depend on condition are obscured, and could be skewing the interpretation of the results (e.g., the visual alpha effect in older adults may be due to one condition only). Can the authors provide more details? If the aim is to present the results simply, then only one condition could be analyzed (e.g., the condition with the most trials). Further, what were the hemisphereic differences of the ROI analysis? This was not shown in the figures. Based on past literature, one might expect that the auditory alpha effect depends on the cued side.

Another issue with the data analysis is the unstructured manner in which the alpha effects and gamma effects were conducted. The gamma analysis arose from a data-driven approach where distributions of t-values in sensor space helped define time and frequency ROIs for the DICS beamformer. The same approach was not given to alpha activity, which was done by simply choosing sensory ROIs. This method bypasses possibly important information about top-down alpha activity in non-sensory areas that could be different between younger and older individuals. I would recommend that the authors use a like-minded data-driven approach to compute time series of alpha activity for all voxels across the brain, and perhaps restrict them to all Brodmann areas to reduce the data space. The cluster-based analysis could be re-analyzed, this time also correcting across spatial location.

The peak alpha analysis also raises concerns on the alpha time series analysis. Clearly there are large individual differences in peak alpha. The qualitative differences in alpha in motor ROI between young and older individuals may account for the effect that was found in high alpha in the time series analysis. What would happen to the time series analysis if it was analyzed at the peak frequency of each participant? Would effects remain? The authors recognize this (l. 444 to 448) and have the opportunity to address it, but do not.

The authors use linear mixed effects models (cited in l 166 and l 249) and reported interactions. However there appeared to be no term for random slope in their model, which leads to higher type I error rates. The authors should repeat the analysis including a term for random slope over the within-subjects factors.

Finally, on l. 85, the authors should describe which data of younger individuals was analyzed in a previous report. The issue of "double dipping" data "or circular analysis is a concern when re-using past data, which can lead to higher false alarm rates if a correction factor is not applied.

Introduction and conceptualization of attention and oscillations:

The characterization of attention consisting of top-down and bottom-up processes is a bit oversimplified. Whereas this conceptualization has enjoyed mainstream focus because it is parsimonious, there are some issues with this view (see Awh et al. 2012; Trends Cogn Sci. 2012 Aug; 16(8): 437–443) such that forms of attention are better described by task goals. For instance "bottom-up" capture (and its neural mechanisms) can be entirely changed depending on the attentional (task) demands placed on the observer. In the present task, bottom-up effects are never fairly assessed because the attentional resources are directed to the cued sound discrimination task---the distractor was never more than a "check" on the listener's top-down ability to focus categorizing the pitch. If a design were to also include a task-switching component where where an unexpected, salient stimulus became the object of focus, then bottom-up systems might be more accurately analyzed.

In a similar vein, the conceptualization of oscillations as fast and slow respectively relating to top-down and bottom-up systems is oversimplified. Alpha oscillations, under the "functional inhibition" hypothesis, do appear to serve a suppressive role in either inhibiting task-irrelevant brain areas or stimuli that are to-be-ignored. However, depending on task and brain region, alpha (power and phase) may also have a positive relationship with neural excitation (Palva and Palva, 2007; Trends in Neurosciences, Vol 30 No 4). Similarly, Gamma oscillations are similarly complex, and the authors also acknowledge that gamma may have a top-down inhibitory role in lines 469-473.

In short it is a bit disingenuous to the breadth of research to simply refer to only top-down and bottom-up mechanisms, and their respective relation to slow and fast oscillations. This may apply to limited circumstances in some research paradigms, but they are not a generalization, and not universally accepted. Importantly, these conceptualizations are not presently supported by the data presented here. I recommend that the authors should re-cast the introduction and discussion so that a balanced perspective is given to the reader.

MINOR POINTS:

l. 13 - "For" appears erroneously at the beginning of this sentence.

l. 77 - Please provide additional details on how scholarship and musical education were quantified and how data were collected.

Section 2.2.1 - I found this section more difficult to follow than it probably was. I recommend starting by describing the trial structure more generally and then referring to the 75% and 25% conditions once the reader grasps the general flow of stimulus presentation.

Section 2.2.2 - Can the authors expand on their choice of tone discrimination? I understand that all participants were able to discriminate the tones, but the closeness of pitch between "high" and "low" tones may be introducing a memory component. For instance, if the authors chose 450 Hz vs 1100 Hz, which ought to still be in the normal hearing region for most participants, the "high" and "low" become easily marked categories. But with pitches close together, they may have to rely on memory of the relative differences established during the initial discrimination task. Such memory demands could impinge on results. During the actual study, what were reaction times and performance measures like for individuals who had increased pitch size differences due to detection problems?

l. 174-175: The concept of "arousal benefit" and "attention capture" are not clear with respect to the condition subtractions. How and why do the authors feel that these contrasts represent arousal and attention, and what evidence supports this?

Figure 2: Has a typo in the word "Distractor"

l. 223 - "beamformer" duplicated in this sentence

l. 258 - The use and description of the "surrogate distractor" is not clear and muddies the understanding of the analysis of gamma activity. Please re-word.

l. 400 - "You" appears erroneously at the beginning of this sentence

l. 405 - Please describe what is meant by "recruitment" here.

l. 444 - Formally (not colloquially) this is not the correct usage of "begging" the question. The authors probably intend to say "raise the question"

**********

6. PLOS authors have the option to publish the peer review history of their article (what does this mean?). If published, this will include your full peer review and any attached files.

If you choose “no”, your identity will remain anonymous but your review may still be made public.

**Do you want your identity to be public for this peer review?** For information about this choice, including consent withdrawal, please see our Privacy Policy.

Reviewer #1: No

Reviewer #2: No

[NOTE: If reviewer comments were submitted as an attachment file, they will be attached to this email and accessible via the submission site. Please log into your account, locate the manuscript record, and check for the action link "View Attachments". If this link does not appear, there are no attachment files to be viewed.]

While revising your submission, please upload your figure files to the Preflight Analysis and Conversion Engine (PACE) digital diagnostic tool, https://pacev2.apexcovantage.com/. PACE helps ensure that figures meet PLOS requirements. To use PACE, you must first register as a user. Registration is free. Then, login and navigate to the UPLOAD tab, where you will find detailed instructions on how to use the tool. If you encounter any issues or have any questions when using PACE, please email us at figures@plos.org. Please note that Supporting Information files do not need this step.

# Author comment: Author response to Decision Letter 0

**DOI:** 10.1371/journal.pone.0229334.r002

1 Nov 2019

Please find detailed responsed to all of the concerns raised by both reviewers in the file entitled: elshafei et al Response to Reviewers.docx

Attachment Submitted filename: elshafei et al Response to Reviewers.docx Click here for additional data file.

# Peer review: Decision Letter 1

**DOI:** 10.1371/journal.pone.0229334.r003

**Authors:** Claude Alain

2 Dec 2019

PONE-D-19-15009R1

Age-Related Modulations of Alpha and Gamma Brain Activities Underlying Anticipation and Distraction

PLOS ONE

Dear Dr. ElShafei,

Thank you for submitting your manuscript to PLOS ONE. After careful consideration, we feel that it has merit but does not fully meet PLOS ONE’s publication criteria as it currently stands. Therefore, we invite you to submit a revised version of the manuscript that addresses the points raised during the review process.

We would appreciate receiving your revised manuscript by Jan 16 2020 11:59PM. When you are ready to submit your revision, log on to https://www.editorialmanager.com/pone/ and select the 'Submissions Needing Revision' folder to locate your manuscript file.

If you would like to make changes to your financial disclosure, please include your updated statement in your cover letter.

To enhance the reproducibility of your results, we recommend that if applicable you deposit your laboratory protocols in protocols.io, where a protocol can be assigned its own identifier (DOI) such that it can be cited independently in the future. For instructions see: http://journals.plos.org/plosone/s/submission-guidelines#loc-laboratory-protocols

Please include the following items when submitting your revised manuscript:

- A rebuttal letter that responds to each point raised by the academic editor and reviewer(s). This letter should be uploaded as separate file and labeled 'Response to Reviewers'.
- A marked-up copy of your manuscript that highlights changes made to the original version. This file should be uploaded as separate file and labeled 'Revised Manuscript with Track Changes'.
- An unmarked version of your revised paper without tracked changes. This file should be uploaded as separate file and labeled 'Manuscript'.

Please note while forming your response, if your article is accepted, you may have the opportunity to make the peer review history publicly available. The record will include editor decision letters (with reviews) and your responses to reviewer comments. If eligible, we will contact you to opt in or out.

We look forward to receiving your revised manuscript.

Kind regards,

Claude Alain

Academic Editor

PLOS ONE

[Note: HTML markup is below. Please do not edit.]

Reviewers' comments:

Reviewer's Responses to Questions

**Comments to the Author**

1. If the authors have adequately addressed your comments raised in a previous round of review and you feel that this manuscript is now acceptable for publication, you may indicate that here to bypass the “Comments to the Author” section, enter your conflict of interest statement in the “Confidential to Editor” section, and submit your "Accept" recommendation.

Reviewer #1: (No Response)

Reviewer #2: (No Response)

**********

2. Is the manuscript technically sound, and do the data support the conclusions?

The manuscript must describe a technically sound piece of scientific research with data that supports the conclusions. Experiments must have been conducted rigorously, with appropriate controls, replication, and sample sizes. The conclusions must be drawn appropriately based on the data presented. 

Reviewer #1: Yes

Reviewer #2: Partly

**********

3. Has the statistical analysis been performed appropriately and rigorously? 

Reviewer #1: Yes

Reviewer #2: Yes

**********

4. Have the authors made all data underlying the findings in their manuscript fully available?

The PLOS Data policy requires authors to make all data underlying the findings described in their manuscript fully available without restriction, with rare exception (please refer to the Data Availability Statement in the manuscript PDF file). The data should be provided as part of the manuscript or its supporting information, or deposited to a public repository. For example, in addition to summary statistics, the data points behind means, medians and variance measures should be available. If there are restrictions on publicly sharing data—e.g. participant privacy or use of data from a third party—those must be specified.

Reviewer #1: Yes

Reviewer #2: No

**********

5. Is the manuscript presented in an intelligible fashion and written in standard English?

PLOS ONE does not copyedit accepted manuscripts, so the language in submitted articles must be clear, correct, and unambiguous. Any typographical or grammatical errors should be corrected at revision, so please note any specific errors here.

Reviewer #1: Yes

Reviewer #2: Yes

**********

6. Review Comments to the Author

Please use the space provided to explain your answers to the questions above. You may also include additional comments for the author, including concerns about dual publication, research ethics, or publication ethics. (Please upload your review as an attachment if it exceeds 20,000 characters)

Reviewer #1: Revision - Age-Related Modulations of Alpha and Gamma Brain Activities Underlying Anticipation and Distraction

I thank the authors for their efforts to respond to my comments. The manuscript has improved substantially. I have still a few comments

Major:

Data visualization and corresponding text: The images and plots are small in some instances and make it hard to identify what the authors describe in the text. For example, Figure 5 includes lots of unnecessary white space and the TFR plots are small. It may also be worth considering to reduce the frequency range plotted in the TFR plots to 20 Hz or 25 Hz as the upper limit. The authors do not report any effects above 15 Hz. The large range makes it difficult to see the relevant details. It was also not clear whether the authors conducted a direction comparison between age groups for the data displayed in Figure 5. It was also not clear why a p<0.1 threshold was chosen for this figure (0.05 in others).

The plots in Figure 6 and 10 could also be made bigger and a color bar could be chosen that allows identifying better where the peak maxima are in the maps. T-values displayed in Figure 9 are small. Are they indeed significant? (z-maps may be better in the future because they are independent of the DFs).

Figure 10 and corresponding text: Was the cue by age group interaction significant? It was not reported. The authors are careful in their wording, which is good. Nevertheless, they may want to be explicit (Page 32: The authors may also explicitly state that the interaction was not significant, if so, to avoid implicit implications).

Minor:

Page 12: The authors write “… Informative NoDIS and of distractor effects on the differences in RTs NoDIS – DIS1 (as a measure of the AROUSAL BENEFIT) or DIS2 – DIS1 (as a measure of DISTRACTION COST) …” It is not explained nor clear why “Arousal Benefit” and “Distraction Cost” would be associated with the different distraction time window. I recommend removing this, since the authors do not come back to it at other places in the manuscript.

Page 14: The authors provide a bit more information about source localization. Some of the information is not fully clear though. Maybe the authors could clearly identify what they used as a volume conductor and what as source model. The two seem somewhat entangled currently. The authors may also want to comment on how they morphed the MNI template was morphed to each individual brain.

Data availability: The data are not made available at this point, but the authors mention that the data are available upon request. This may be sufficient for PLoS ONE, I only wanted to raise the issue, because it was one of the points in the drop-down menu.

Reviewer #2: I thank the authors for addressing my concerns. The changes the authors have made have been clearly outlined, and I also thank the authors for their additional efforts to articulate the comments from both reviewers, providing supplemental flow charts and plots supporting their responses. My opinion is that the quality of the manuscript has been greatly improved. Below I have additional comments based on the new results and changes.

1. With the results now fully understood, there is an outstanding concern with the stimulus design that impacts the interpretation of the results. Stimulus levels for both target and distractor sounds were set based upon the threshold for the target stimuli. The target stimuli were between 512 to three semitones above 575 Hz. However, the distractor stimuli were complex sounds (phone rings, alarms, etc) which likely have acoustic spectra that reach much higher frequencies (if the authors could supply those, this would be helpful). Older adults commonly have elevated thresholds at frequencies higher than the target stimulus. Thus, the sensation level of the distractor tone, which was based upon the threshold of the target tone, was most likely lower for older adults than it was for younger adults. No audiograms were supplied (or apparently collected) which could indicate otherwise. If older adults received less salient (i.e., loud) distractor stimuli than younger adults, the difference has consequences for the interpretation of the "bottom-up" effect of the distractor as a function of age. Older adults had evidence of reduced activation of gamma activity in lateral prefrontal regions compared to younger adults. Because of varying sensation levels in older adults, it cannot be determined if this effect is due to aging or lower sensation level of the distractor due to hearing loss. At minimum, this is a major caveat that should condition the interpretation provided by the authors. While there does seem to be evidence of reduced top-down "filtering" via weaker modulation of visual alpha, the role of the "bottom-up" attention network remains equivocal. The conclusions of the study (e.g., entire section 4.3, 4.4, opening of section 4) likely need to be adjusted.

2. As a brief comment, was there an attempt to predict inter-individual behavioral performance from the neural variables? This might strengthen the arguments for or against the balance of top-down filtering via alpha or modulation of distractor processing in attention networks via gamma.

3. It remains unclear to me how the DIS1 and DIS2 time windows were determined. Based on a reply to the other reviewer, the authors establish that there is not a linear increase of RT as a function of the distractor time. However the binning of RT by 39 ms steps (instead of plotting a continuous function) obscures any obvious pivot points as a function of time that suggest that later distractors are of a separate process. In other words, Figure 6 is not convincing as to support their DIS1/2 windows. Bins 3,4,and 5 on the abscissa could conceivably belong to either the early or the late windows. This has implications for both the analysis windows for neural variables and also behavior, and it would benefit the paper if there was a stronger motivation.

4. This is more of a stylistic suggestion---axis labels and text in some of the figures could be enlarged. For instance, Figures 5 and 9 have very small text for axis ticks, but other figures (behavioral ones) enjoy larger text.

5. As a final comment, it would be helpful for the manuscript if the authors could go through carefully and adjust the very minor grammatical and style errors that remain throughout the manuscript. Some examples:

l. 43: there should be a hyphen, not an n-dash, between "top" and "down"

l. 87-88 ranges should be separated by n-dashes, not m-dashes.

l. 110: no hyphen is needed between "grey" and "background.

l. 112: "only" is not needed in this sentence.

l. 113-115: after "DIS2," use the word "and" or use semicolons to properly break up the clauses in this sentence

l. 120: "the" before "distracting sound"

l. 135: "the" before "informative cue"

This list is not exhaustive.

**********

7. PLOS authors have the option to publish the peer review history of their article (what does this mean?). If published, this will include your full peer review and any attached files.

If you choose “no”, your identity will remain anonymous but your review may still be made public.

**Do you want your identity to be public for this peer review?** For information about this choice, including consent withdrawal, please see our Privacy Policy.

Reviewer #1: No

Reviewer #2: No

[NOTE: If reviewer comments were submitted as an attachment file, they will be attached to this email and accessible via the submission site. Please log into your account, locate the manuscript record, and check for the action link "View Attachments". If this link does not appear, there are no attachment files to be viewed.]

While revising your submission, please upload your figure files to the Preflight Analysis and Conversion Engine (PACE) digital diagnostic tool, https://pacev2.apexcovantage.com/. PACE helps ensure that figures meet PLOS requirements. To use PACE, you must first register as a user. Registration is free. Then, login and navigate to the UPLOAD tab, where you will find detailed instructions on how to use the tool. If you encounter any issues or have any questions when using PACE, please email us at figures@plos.org. Please note that Supporting Information files do not need this step.

# Author comment: Author response to Decision Letter 1

**DOI:** 10.1371/journal.pone.0229334.r004

10 Jan 2020

You can find a detailed account of how we have responded to the reviewers' comments in the “elshafei et al second round Response to Reviewers.docx”:

We thank the reviewers for their positive feedback on our revised manuscript. We truly believe that, based upon their comments, the manuscript has greatly improved. We hope that this revised version would address all of the reviewers’ concerns. Below, we have laid out the reviewers’ comments (in blue font) along with our replies in black. We have taken the liberty to number the reviewer’s comments so that they are easier to refer to. In the revised manuscript, modifications are highlighted in yellow.

Reviewer #1: Revision - Age-Related Modulations of Alpha and Gamma Brain Activities Underlying Anticipation and Distraction

I thank the authors for their efforts to respond to my comments. The manuscript has improved substantially. I have still a few comments

Major:

Data visualization and corresponding text: 

1. The images and plots are small in some instances and make it hard to identify what the authors describe in the text. For example, Figure 5 includes lots of unnecessary white space and the TFR plots are small. It may also be worth considering to reduce the frequency range plotted in the TFR plots to 20 Hz or 25 Hz as the upper limit. The authors do not report any effects above 15 Hz. The large range makes it difficult to see the relevant details. It was also not clear whether the authors conducted a direction comparison between age groups for the data displayed in Figure 5. It was also not clear why a p<0.1 threshold was chosen for this figure (0.05 in others). The plots in Figure 6 and 10 could also be made bigger and a color bar could be chosen that allows identifying better where the peak maxima are in the maps. T-values displayed in Figure 9 are small. Are they indeed significant? (z-maps may be better in the future because they are independent of the DFs). Figure 10 and corresponding text: Was the cue by age group interaction significant? It was not reported. The authors are careful in their wording, which is good. Nevertheless, they may want to be explicit (Page 32: The authors may also explicitly state that the interaction was not significant, if so, to avoid implicit implications).

We apologize for these inaccuracies. We have revised all of our figures, increased font size and reduced white space.

• For figure 5: we have reduced the frequency range to 25Hz. Only, for that figure, a p<0.1 threshold was chosen to elucidate a trending effect of great relevance i.e. distinct (low/high) alpha sub-bands support distinct (facilitatory/inhibitory) top-down attentional mechanisms, in the younger group.

• For figure 6 and 10, we have adapted the color bars to highlight the peak maxima. We also changed the text on the figures and the captions since no direction comparison between groups has been performed.

• We agree with the reviewer that the t-values in Figure 6 seem fairly low. This is due to (1) averaging across time, frequency, or sensors in relatively large space-time-frequency windows, and (2) relative low signal-to-noise ratio of high frequency activity. However, these values are indeed significant as indicated by the p-value of the cluster (p < 0.001). 

• In figure 10, we do not directly test for a cue by group interaction, due to the limitations of cluster-based permutation tests. Thus, we perform the similar test (informative versus uninformative) for each group separately. A note has been added to the manuscript to clarify this point.

Minor:

2. Page 12: The authors write “… Informative NoDIS and of distractor effects on the differences in RTs NoDIS – DIS1 (as a measure of the AROUSAL BENEFIT) or DIS2 – DIS1 (as a measure of DISTRACTION COST) …” It is not explained nor clear why “Arousal Benefit” and “Distraction Cost” would be associated with the different distraction time window. I recommend removing this, since the authors do not come back to it at other places in the manuscript.

We respectfully disagree with the reviewer, but we do come back to that distinction in the Results (section 3.2.1) and the Discussion (section 4.1) for which we now provide a schematic representation in Figure1 below. Thus, we prefer not to remove that sentence. In addition, the justification for using these differences can be found in the cited references: 

[1] Bidet-Caulet A, Bottemanne L, Fonteneau C, Giard M-H, Bertrand O. Brain Dynamics of Distractibility: Interaction Between Top-Down and Bottom-Up Mechanisms of Auditory Attention. Brain Topogr. 2014;28(3):423–36. 

[2] Masson R, Bidet-Caulet A. Fronto-central P3a to distracting sounds: An index of their arousing properties. NeuroImage. 2019 Jan 15;185:164–80. 

Figure1. Schematic representation of the behavioral effects of distracting sounds in the Competitive Attention Test.

3. Page 14: The authors provide a bit more information about source localization. Some of the information is not fully clear though. Maybe the authors could clearly identify what they used as a volume conductor and what as source model. The two seem somewhat entangled currently. The authors may also want to comment on how they morphed the MNI template was morphed to each individual brain.

We apologize for the lack all of clarity. We now report additional information (e.g. tapers, regularization factor) for our source localization methods. In addition, we clearly state that:

“For each participant, an anatomically realistic single-shell headmodel (volume conduction model) based on the cortical surface was generated from individual head shape. A grid (source model) with 0.5-cm resolution was created using an MNI template, and then morphed (adjusted) into the brain volume of each participant using non-linear transformation. This procedure ensures that each grid-point would represent the same anatomical label across all subjects but with different subject-specific head positions.”

4. Data availability: The data are not made available at this point, but the authors mention that the data are available upon request. This may be sufficient for PLoS ONE, I only wanted to raise the issue, because it was one of the points in the drop-down menu.

We thank the reviewers for raising this point. This point has been addressed directly with the editor. 

Reviewer #2: I thank the authors for addressing my concerns. The changes the authors have made have been clearly outlined, and I also thank the authors for their additional efforts to articulate the comments from both reviewers, providing supplemental flow charts and plots supporting their responses. My opinion is that the quality of the manuscript has been greatly improved. Below I have additional comments based on the new results and changes.

1. With the results now fully understood, there is an outstanding concern with the stimulus design that impacts the interpretation of the results. Stimulus levels for both target and distractor sounds were set based upon the threshold for the target stimuli. The target stimuli were between 512 to three semitones above 575 Hz. However, the distractor stimuli were complex sounds (phone rings, alarms, etc) which likely have acoustic spectra that reach much higher frequencies (if the authors could supply those, this would be helpful). Older adults commonly have elevated thresholds at frequencies higher than the target stimulus. Thus, the sensation level of the distractor tone, which was based upon the threshold of the target tone, was most likely lower for older adults than it was for younger adults. No audiograms were supplied (or apparently collected) which could indicate otherwise. If older adults received less salient (i.e., loud) distractor stimuli than younger adults, the difference has consequences for the interpretation of the "bottom-up" effect of the distractor as a function of age. Older adults had evidence of reduced activation of gamma activity in lateral prefrontal regions compared to younger adults. Because of varying sensation levels in older adults, it cannot be determined if this effect is due to aging or lower sensation level of the distractor due to hearing loss. At minimum, this is a major caveat that should condition the interpretation provided by the authors. While there does seem to be evidence of reduced top-down "filtering" via weaker modulation of visual alpha, the role of the "bottom-up" attention network remains equivocal. The conclusions of the study (e.g., entire section 4.3, 4.4, opening of section 4) likely need to be adjusted.

This is a very relevant remark. As shown in Figure2, the acoustic spectra for some of the distracting sounds reach high frequencies while some are restricted to rather low frequencies (below 1500 Hz). 

As shown in Figure3, we split the distracting sounds into 18 low and 18 high frequency sounds (using 1500Hz as the border) and re-run the behavioral analysis on median reaction times (general linear mixed models) with three factors (age, distracting-sound delay and distracting-sound frequency). Similarly to our original analysis, we found a main effect of distracting-sound delay (F(2,27) = 118.39, p < 0.001, η2 = 0.53) on reaction times and a significant interaction between age and distractor sound delay (F(1,27) = 16.95, p < 0.001, η2 = 0.17). However, we found neither a significant main effect of significant distractor sound frequency (F(2,27) = 0.13, p = 0.71, η2 = 0.002), nor a significant interaction between age and distractor sound frequency (F(1,27) = 1.93, p = 0.16, η2 = 0.024). 

These results were confirmed by a Bayesian repeated-measure ANOVA with the same factors. This analysis showed that the best model accounting for the data is the one with the main effect of distracting-sound delay and the interaction between age and distracting-sound delay (BF10 = 4.1e+14). There was decisive evidence for the effect of distracting-sound delay (BFinclusion=9.5e +13) and the interaction between age and distracting-sound delay (BFinclusion=232.4), strong evidence for the effect of age (BFinclusion=77.2), no evidence for the interaction between distracting-sound frequency and position (BFinclusion=1.0), the interaction between age and distractor sound frequency (BFinclusion=0.6), and the triple interaction (BFinclusion=0.6), and positive evidence against the effect of distracting-sound frequency (BFinclusion=0.3).

These two analysis strongly suggest that, irrespective of the frequency content of distracting sounds, elderly participants show longer reaction times after late distracting sounds. In other words, elderly show the same enhanced distraction effect irrespective of the frequency content of distracting sounds. Therefore, it seems quite unlikely that results observed at the brain level are due to differences in sensation level between elderly and young participants.

Figure2. Acoustic spectra for the 40 distracting sounds used for the experiment.

Figure3. Median reaction times in each group according to distracting-sound delay and frequency conditions.

2. As a brief comment, was there an attempt to predict inter-individual behavioral performance from the neural variables? This might strengthen the arguments for or against the balance of top-down filtering via alpha or modulation of distractor processing in attention networks via gamma.

This is a very good suggestion. We have indeed computed correlations between behavioral performances (distraction effect and cue benefit) and neural variables (alpha and gamma activity). However, no test reached statistical significance when performed on a whole brain level i.e. with no pre-defined regions of interest. However, upon defining four regions of interest (lateral prefrontal cortex, visual, auditory and motor cortices), we have found significant correlations only between the distraction effect and:

� Gamma activity in the lateral prefrontal cortex and (rspearman= - 0.37, p = 0.04).

� Alpha activity (0.6-1 s, post-cue) in the visual cortex, i.e. marker of top-down suppression (rspearman= - 0.43, p = 0.02).

In addition, the aforementioned activities were also significantly correlated (rspearman= 0.41, p = 0.02). Interestingly, the distraction effect did not correlate with alpha activity in the auditory nor motor cortex, i.e. markers of top-down facilitation.

Figure4. Scatter plots of behavioral distraction effect against alpha activity in the visual cortex and gamma activity in the lateral prefrontal cortex.

3. It remains unclear to me how the DIS1 and DIS2 time windows were determined. Based on a reply to the other reviewer, the authors establish that there is not a linear increase of RT as a function of the distractor time. However the binning of RT by 39 ms steps (instead of plotting a continuous function) obscures any obvious pivot points as a function of time that suggest that later distractors are of a separate process. In other words, Figure 6 is not convincing as to support their DIS1/2 windows. Bins 3,4,and 5 on the abscissa could conceivably belong to either the early or the late windows. This has implications for both the analysis windows for neural variables and also behavior, and it would benefit the paper if there was a stronger motivation.

We apologize if the choice for distractor delays remains unclear. The binning was done in such a way to encompass sufficient trials since trials with distractors constitute only 25% of trials. Thus, plotting the data in a continuous manner would not be informative and rather noisy. 

As shown in Figure1 (above), the dissociation between DIS1 and DIS2 time-windows is only relevant for behavior analysis, since the impact of the two phenomena triggered by a distracting sound (arousal burst and distraction) on the reaction times to target differ according to the distractor-target delay. This dissociation provides a good approximation at the behavioral level of the strength of the arousal burst and distraction phenomena. At the brain level, this dissociation would be rather meaningless, since both phenomena are triggered irrespective of the distractor-target delay. Therefore, the brain analyses have been performed on the average of all distracting sounds.

4. This is more of a stylistic suggestion---axis labels and text in some of the figures could be enlarged. For instance, Figures 5 and 9 have very small text for axis ticks, but other figures (behavioral ones) enjoy larger text.

We thank the reviewer for this suggestion. We have revised all of our figures, increased font size and reduced white space

5. As a final comment, it would be helpful for the manuscript if the authors could go through carefully and adjust the very minor grammatical and style errors that remain throughout the manuscript. Some examples:

l. 43: there should be a hyphen, not an n-dash, between "top" and "down"

l. 87-88 ranges should be separated by n-dashes, not m-dashes.

l. 110: no hyphen is needed between "grey" and "background.

l. 112: "only" is not needed in this sentence.

l. 113-115: after "DIS2," use the word "and" or use semicolons to properly break up the clauses in this sentence

l. 120: "the" before "distracting sound"

l. 135: "the" before "informative cue"

This list is not exhaustive.

We apologize for these mistakes. We have now fully proofread the manuscript for such errors.

Attachment Submitted filename: elshafei et al second round Response to Reviewers.docx Click here for additional data file.

# Peer review: Decision Letter 2

**DOI:** 10.1371/journal.pone.0229334.r005

**Authors:** Claude Alain

27 Jan 2020

PONE-D-19-15009R2

Age-Related Modulations of Alpha and Gamma Brain Activities Underlying Anticipation and Distraction

PLOS ONE

Dear Dr. ElShafei,

Thank you for submitting your manuscript to PLOS ONE. After careful consideration, we feel that it has merit but does not fully meet PLOS ONE’s publication criteria as it currently stands. Therefore, we invite you to submit a revised version of the manuscript that addresses the points raised during the review process.

The requested revisions are minor and given your response I may not send the manuscript out for review and instead review it myself. 

We would appreciate receiving your revised manuscript by Mar 12 2020 11:59PM. When you are ready to submit your revision, log on to https://www.editorialmanager.com/pone/ and select the 'Submissions Needing Revision' folder to locate your manuscript file.

If you would like to make changes to your financial disclosure, please include your updated statement in your cover letter.

To enhance the reproducibility of your results, we recommend that if applicable you deposit your laboratory protocols in protocols.io, where a protocol can be assigned its own identifier (DOI) such that it can be cited independently in the future. For instructions see: http://journals.plos.org/plosone/s/submission-guidelines#loc-laboratory-protocols

Please include the following items when submitting your revised manuscript:

- A rebuttal letter that responds to each point raised by the academic editor and reviewer(s). This letter should be uploaded as separate file and labeled 'Response to Reviewers'.
- A marked-up copy of your manuscript that highlights changes made to the original version. This file should be uploaded as separate file and labeled 'Revised Manuscript with Track Changes'.
- An unmarked version of your revised paper without tracked changes. This file should be uploaded as separate file and labeled 'Manuscript'.

Please note while forming your response, if your article is accepted, you may have the opportunity to make the peer review history publicly available. The record will include editor decision letters (with reviews) and your responses to reviewer comments. If eligible, we will contact you to opt in or out.

We look forward to receiving your revised manuscript.

Kind regards,

Claude Alain

Academic Editor

PLOS ONE

[Note: HTML markup is below. Please do not edit.]

Reviewers' comments:

Reviewer's Responses to Questions

**Comments to the Author**

1. If the authors have adequately addressed your comments raised in a previous round of review and you feel that this manuscript is now acceptable for publication, you may indicate that here to bypass the “Comments to the Author” section, enter your conflict of interest statement in the “Confidential to Editor” section, and submit your "Accept" recommendation.

Reviewer #1: All comments have been addressed

Reviewer #2: (No Response)

**********

2. Is the manuscript technically sound, and do the data support the conclusions?

The manuscript must describe a technically sound piece of scientific research with data that supports the conclusions. Experiments must have been conducted rigorously, with appropriate controls, replication, and sample sizes. The conclusions must be drawn appropriately based on the data presented. 

Reviewer #1: Yes

Reviewer #2: Partly

**********

3. Has the statistical analysis been performed appropriately and rigorously? 

Reviewer #1: Yes

Reviewer #2: Yes

**********

4. Have the authors made all data underlying the findings in their manuscript fully available?

The PLOS Data policy requires authors to make all data underlying the findings described in their manuscript fully available without restriction, with rare exception (please refer to the Data Availability Statement in the manuscript PDF file). The data should be provided as part of the manuscript or its supporting information, or deposited to a public repository. For example, in addition to summary statistics, the data points behind means, medians and variance measures should be available. If there are restrictions on publicly sharing data—e.g. participant privacy or use of data from a third party—those must be specified.

Reviewer #1: Yes

Reviewer #2: Yes

**********

5. Is the manuscript presented in an intelligible fashion and written in standard English?

PLOS ONE does not copyedit accepted manuscripts, so the language in submitted articles must be clear, correct, and unambiguous. Any typographical or grammatical errors should be corrected at revision, so please note any specific errors here.

Reviewer #1: Yes

Reviewer #2: Yes

**********

6. Review Comments to the Author

Please use the space provided to explain your answers to the questions above. You may also include additional comments for the author, including concerns about dual publication, research ethics, or publication ethics. (Please upload your review as an attachment if it exceeds 20,000 characters)

Reviewer #1: I thank the authors for their efforts. I have no further comments.

Reviewer #2: I thank the authors for addressing my comments and providing visualization for an explanation. They have been clear and concise in the response. I have only one remaining concern:

The authors wrote: "These two analysis strongly suggest that, irrespective of the frequency content of distracting sounds, elderly participants show longer reaction times after late distracting sounds"

The above comment in reference to a potential confound with sensation level for distracting sounds for the older adults. I appreciate the detailed analysis showing that the stimuli with acoustically higher frequencies did not have an observable effect on behavior. However my comment originally was concerned with the effect on gamma activity, which was not addressed in the authors' response.

In a separate comment the authors found that gamma activity was only weakly correlated with the behavioral distractor effect, accounting for very little of the variance. Thus the absence of an effect on behavioral data does not preclude an effect on the neural data, as well as the interpretation of this effect.

Unless otherwise supported by data, the caveat warrants a note of limitation in the discussion of gamma results in older adults, and the discussion of the results in section 4.3 should be tempered.

**********

7. PLOS authors have the option to publish the peer review history of their article (what does this mean?). If published, this will include your full peer review and any attached files.

If you choose “no”, your identity will remain anonymous but your review may still be made public.

**Do you want your identity to be public for this peer review?** For information about this choice, including consent withdrawal, please see our Privacy Policy.

Reviewer #1: No

Reviewer #2: No

[NOTE: If reviewer comments were submitted as an attachment file, they will be attached to this email and accessible via the submission site. Please log into your account, locate the manuscript record, and check for the action link "View Attachments". If this link does not appear, there are no attachment files to be viewed.]

While revising your submission, please upload your figure files to the Preflight Analysis and Conversion Engine (PACE) digital diagnostic tool, https://pacev2.apexcovantage.com/. PACE helps ensure that figures meet PLOS requirements. To use PACE, you must first register as a user. Registration is free. Then, login and navigate to the UPLOAD tab, where you will find detailed instructions on how to use the tool. If you encounter any issues or have any questions when using PACE, please email us at figures@plos.org. Please note that Supporting Information files do not need this step.

# Author comment: Author response to Decision Letter 2

**DOI:** 10.1371/journal.pone.0229334.r006

30 Jan 2020

We thank both reviewers for their positive feedback on our revised manuscript. Below, we have laid out the reviewers’ remaining comments (in blue font) along with our reply in black. In the revised manuscript, modifications are highlighted in yellow.

Reviewer #2: 

I thank the authors for addressing my comments and providing visualization for an explanation. They have been clear and concise in the response. I have only one remaining concern:

The authors wrote: "These two analysis strongly suggest that, irrespective of the frequency content of distracting sounds, elderly participants show longer reaction times after late distracting sounds". The above comment in reference to a potential confound with sensation level for distracting sounds for the older adults. 

I appreciate the detailed analysis showing that the stimuli with acoustically higher frequencies did not have an observable effect on behavior. However my comment originally was concerned with the effect on gamma activity, which was not addressed in the authors' response. In a separate comment the authors found that gamma activity was only weakly correlated with the behavioral distractor effect, accounting for very little of the variance. Thus the absence of an effect on behavioral data does not preclude an effect on the neural data, as well as the interpretation of this effect. Unless otherwise supported by data, the caveat warrants a note of limitation in the discussion of gamma results in older adults, and the discussion of the results in section 4.3 should be tempered.

We understand the reviewer’s concern. Accordingly, we have addressed such limitation in the manuscript: 

“It is important to note that, in the present study, distracting sounds were complex sounds with acoustic spectra reaching high frequencies where older participants can display altered sensation levels. These potential differences in sensation levels between groups could result in neural activity differences. However, we believe that differences in sensation levels, related to peripheral damage, would have mostly manifested as differences in gamma activation in the ventral BU attentional network, in particular in the auditory cortices, rather than in lateral prefrontal regions. Due to the relatively low number of trials with distracting sounds and low signal-to-noise ratio of gamma activity, we were not able to factor spectral content of distracting sounds in our gamma analyses. However, the behavioral analysis on median reaction times (supplementary figure 5) according to the low or high spectral content of the distracting sounds showed neither a significant main effect of spectral content, nor a significant interaction between age and spectral content. While the absence of an effect on behavioral data does not preclude an effect on the neural data, we believe that potential differences in sensation levels is quite unlikely to explain differences in gamma activation between groups.”

Attachment Submitted filename: elshafei et al third round Response to Reviewers.docx Click here for additional data file.

# Editor report: Decision Letter 3

**DOI:** 10.1371/journal.pone.0229334.r007

**Authors:** Claude Alain

5 Feb 2020

Age-Related Modulations of Alpha and Gamma Brain Activities Underlying Anticipation and Distraction

PONE-D-19-15009R3

Dear Dr. ElShafei,

We are pleased to inform you that your manuscript has been judged scientifically suitable for publication and will be formally accepted for publication once it complies with all outstanding technical requirements.

Within one week, you will receive an e-mail containing information on the amendments required prior to publication. When all required modifications have been addressed, you will receive a formal acceptance letter and your manuscript will proceed to our production department and be scheduled for publication.

Shortly after the formal acceptance letter is sent, an invoice for payment will follow. To ensure an efficient production and billing process, please log into Editorial Manager at https://www.editorialmanager.com/pone/, click the "Update My Information" link at the top of the page, and update your user information. If you have any billing related questions, please contact our Author Billing department directly at authorbilling@plos.org.

If your institution or institutions have a press office, please notify them about your upcoming paper to enable them to help maximize its impact. If they will be preparing press materials for this manuscript, you must inform our press team as soon as possible and no later than 48 hours after receiving the formal acceptance. Your manuscript will remain under strict press embargo until 2 pm Eastern Time on the date of publication. For more information, please contact onepress@plos.org.

With kind regards,

Claude Alain

Academic Editor

PLOS ONE

Additional Editor Comments (optional):

Reviewers' comments:

# Editor report: Acceptance letter

**DOI:** 10.1371/journal.pone.0229334.r008

**Authors:** Claude Alain

7 Feb 2020

PONE-D-19-15009R3 

Age-Related Modulations of Alpha and Gamma Brain Activities Underlying Anticipation and Distraction 

Dear Dr. ElShafei:

I am pleased to inform you that your manuscript has been deemed suitable for publication in PLOS ONE. Congratulations! Your manuscript is now with our production department. 

If your institution or institutions have a press office, please notify them about your upcoming paper at this point, to enable them to help maximize its impact. If they will be preparing press materials for this manuscript, please inform our press team within the next 48 hours. Your manuscript will remain under strict press embargo until 2 pm Eastern Time on the date of publication. For more information please contact onepress@plos.org.

For any other questions or concerns, please email plosone@plos.org. 

Thank you for submitting your work to PLOS ONE.

With kind regards,

PLOS ONE Editorial Office Staff

on behalf of

Dr. Claude Alain 

Academic Editor

PLOS ONE
